# The interplay of transcriptional coregulator NUPR1 with SREBP1 promotes hepatocellular carcinoma progression via upregulation of lipogenesis

**DOI:** 10.1038/s41420-022-01213-z

**Published:** 2022-10-28

**Authors:** Yongjia Wang, Yuqin Zhang, Zixuan Wang, Lu Yu, Keli Chen, Yuwen Xie, Yang Liu, Weijie Liang, Yilin Zheng, Yizhi Zhan, Yi Ding

**Affiliations:** 1 Department of Radiation Oncology, Nanfang Hospital, Southern Medical University, Guangzhou, Guangdong province China; 2 Department of General Medicine, The First Affiliated Hospital of Jinan University, Guangzhou, Guangdong province China; 3 The First School of Clinical Medicine, Southern Medical University, Guangzhou, Guangdong province China; 4 Huiqiao Medical Center, Nanfang Hospital, Southern Medical University, Guangzhou, Guangdong province China; 5 Department of General Surgery, Nanfang Hospital, Southern Medical University, Guangzhou, Guangdong province China

**Keywords:** Oncogenes, Cancer metabolism, Gastrointestinal cancer

## Abstract

Nuclear protein 1 (NUPR1) is a transcriptional coregulator that has been implicated in the development of various cancer types. In addition, de novo fatty acid synthesis plays a pivotal role in hepatocellular carcinoma (HCC) development. However, little is currently known on the role of NUPR1 in hepatocellular carcinoma. In this study, bioinformatics analysis was conducted to analyze the expression level, prognosis value and enriched pathways of NUPR1 in Liver Hepatocellular Carcinoma (LIHC). We found that NUPR1 was significantly upregulated in human hepatocellular carcinoma cells compared with normal hepatocytes from LIHC patients in TCGA cohorts and our patients. Kaplan–Meier analysis and COX proportional hazard progression model showed that high expression of NUPR1 was correlated with a poor prognosis of LIHC patients. CCK-8, EdU and colony formation assays were performed to explore the effect of NUPR1 on the proliferation of HCC cells, then wound healing and transwell migration assays were performed to evaluate the effects of NUPR1 on cell migration. Furthermore, subcutaneous xenograft models were established to study tumor growth. Results showed that NUPR1 overexpression correlated with a highly proliferative and aggressive phenotype. In addition, NUPR1 knockdown significantly inhibited hepatocellular carcinoma cell proliferation and migration in vitro and hindered tumorigenesis in vivo. Mechanistically, endogenous NUPR1 could interact with sterol regulatory element binding protein 1 (SREBP1) and upregulated lipogenic gene expression of fatty acid synthase (FASN), resulting in the accumulation of lipid content. Moreover, pharmacological or genetic blockade of the NUPR1-SREBP1/FASN pathway enhanced anticancer activity in vitro and in vivo. Overall, we identified a novel function of NUPR1 in regulating hepatocellular carcinoma progression via modulation of SREBP1-mediated de novo lipogenesis. Targeting NUPR1-SREBP1/FASN pathway may be a therapeutic alternative for hepatocellular carcinoma.

## Introduction

Hepatocellular carcinoma (HCC) is the sixth most prevalent cancer and the third-leading cause of cancer-related death worldwide, with increasing incidence and few effective treatment options available [1]. The majority of HCC occurs in the setting of chronic liver diseases, such as infection with hepatitis B or hepatitis C and non-alcoholic steatohepatitis [2]. The liver is a central metabolic organ within the human body that has been established to play a central role in the synthesis, storage and degradation of lipids. Accumulating evidence shows that lipid metabolism is altered in human liver tumors, which upregulates de novo lipogenesis to ensure that proliferating cells have access to extra lipids for membrane biogenesis, energy source, signaling lipid molecules and post-translational modifications [3, 4]. In addition, aberrant expression and activity of key enzymes involved in de novo fatty acid synthesis, such as fatty acid synthase (FASN) and stearoyl-CoA desaturase 1 (SCD1), have been identified to contribute to HCC development [5]. Sterol response element-binding protein 1 (SREBP1), a master transcription factor of de novo lipogenesis and lipid homeostasis, has been reported to induce lipogenic reprogramming of tumor cells and provide a critical link between oncogenic signaling and tumor metabolism [6]. The cleavage of precursor SREBP1 yields the mature SREBP1 (mSREBP1) product. Once mature, active SREBP1 translocates to the nucleus and transactivates the expression of its target genes, such as FASN and SCD1 [7]. Nevertheless, SREBP1 requires additional co-regulatory transcription factors to regulate promoters properly [8].

Nuclear protein 1 (NUPR1/p8/COM1) is a transcriptional coregulator and a multifunctional stress-associated protein that has recently elicited great attention for its role in several protumorigenic processes in various cancer types, including cell growth, migration, invasion, drug resistance, ferroptosis, angiogenesis and mitochondrial respiratory [9–11]. Elevated expression of NUPR1 has been associated with a high-fat diet, and it has been suggested that NUPR1 can protect tissues from cell injury in the context of obesity and a high-fat diet [12, 13]. Given that the liver is the most important organ involved in lipid synthesis and metabolism and NUPR1 is related to lipid metabolism, we sought to examine whether NUPR1 could affect HCC progression by altering lipid metabolism.

In the present study, we found that NUPR1 could interact with mSREBP1, indicating that NUPR1 acts as a co-regulatory transcription factor of SREBP1. Moreover, we found that NUPR1 could promote the malignant potential of liver cancer cells by facilitating nuclear translocation of the transcriptionally active form of SREBP1 and transactivating target genes FASN and SCD1, resulting in lipid accumulation. In addition, pharmacological or genetic blockade of the NUPR1-SREBP1/FASN pathway enhanced anticancer activity in vivo and in vitro.

## Results

### NUPR1 is upregulated in LIHC tissues and correlates with poor survival

**Fig. 1:**
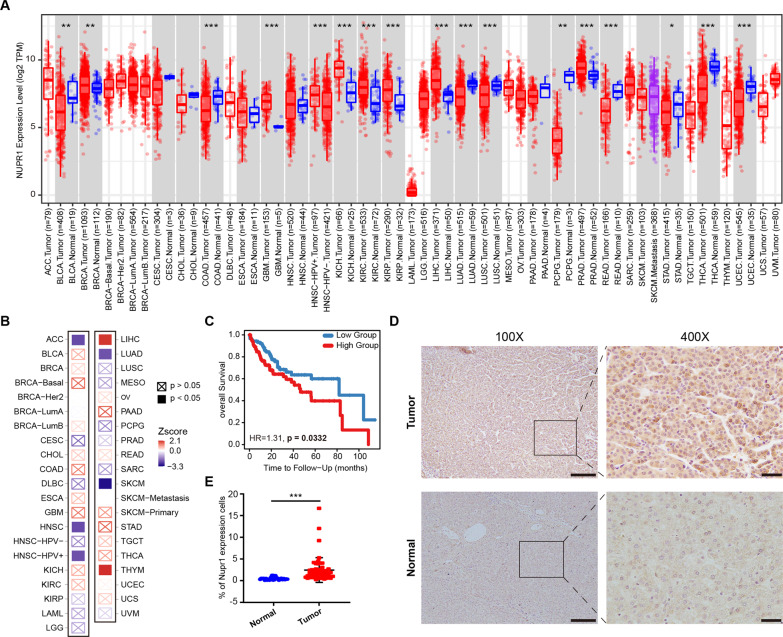
Expression level and clinical values of NUPR1 in LIHC. **A** mRNA expression difference of NUPR1 between normal and tumor samples using TIMER2.0 database. **B** Cox proportional hazard model to evaluate the outcome significance of NUPR1 via TIMER2.0 database, adjusted by clinical stage. **C** Kaplan–Meier curves for LIHC subjects with Low Group versus High Group using TIMER2.0 database. **D**, **E** Representative images (left panels, scale bars 100 µm and right panels, scale bars 20 µm) and quantification of immunohistochemistry staining are presented for NUPR1 protein in clinical normal liver (*n* = 24) and HCC tissues (*n* = 50).

To preliminarily investigate the transcriptional expression level of NUPR1 in cancers, we analyzed a TCGA pan-cancer cohort. We found that NUPR1 was upregulated in multiple cancer types, including LIHC (liver hepatocellular carcinoma (HCC)), BRCA (breast invasive carcinoma), GBM (glioblastoma multiforme), KICH (kidney chromophobe), KIRC (kidney renal clear cell carcinoma), KIRP (kidney renal papillary cell carcinoma) and PRAD (prostate adenocarcinoma) (Fig. 1A). After stratifying by clinical stage, Cox proportional hazard model was used to evaluate the significance of NUPR1 on patient outcomes. We found that NUPR1 was a risk factor for LIHC and THYM (Thymoma) (Fig. 1B). In addition, the Kaplan–Meier Curve showed that NUPR1 expression was associated with LIHC patient survival, as the low-expression group had significantly better 10-year overall survival than the high-expression group (*p* = 0.033 Fig. 1C). To further substantiate the clinical relevance of our findings, we analyzed the NUPR1 expression in tissues from LIHC patients, which showed that the expression of NUPR1 was significantly increased in tumor tissues compared to normal tissues (Fig. 1D, E). These results provide compelling evidence that NUPR1 may be related to LIHC progression.

### NUPR1 promotes HCC cells proliferation in vitro and induces tumor growth in vivo

**Fig. 2:**
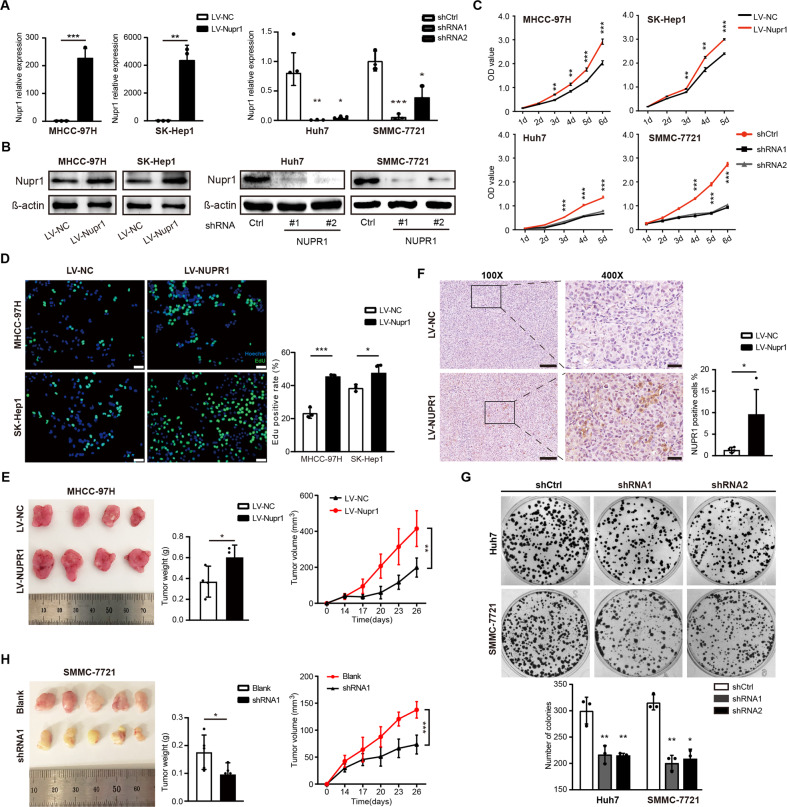
NUPR1 promotes HCC cell proliferation in vitro and induces tumor growth in vivo. **A**, **B** Efficiency of overexpression and knockdown against human NUPR1 was verified by qRT-PCR assay (a) and western blot (b) and β-actin as an internal control. **C** The impact of aberrant NUPR1 expression on cell proliferation among HCC cells was detected by CCK8 assay. **D** The proliferation ability of MHCC-97H and SK-Hep1 cells overexpressed NUPR1 was detected by EdU assays, scale bars represent 20 µm. **E** Effects of NUPR1 on HCC xenograft tumor growth in vivo. Left: Representative images of tumors in nude mice after subcutaneous injection of MHCC-97H cells with LV-NUPR1 and LV-NC (*n* = 4). Middle: Column scatter plot of the xenograft tumor weight. Right: Tumor growth curve was plotted using xenograft tumor volume data. **F** NUPR1 expression in MHCC-97H xenograft tumor was detected by immunohistochemistry (left panels, scale bars 100 µm and right panels, scale bars 20 µm). **G** The images and statistical analyses display the colony formation of Huh7 and SMMC-7721 transfected with sh-NUPR1. **H** Xenograft growth in nude mice injected with SMMC-7721 and SMMC-7721-shNUPR1 (*n* = 5). Tumor photographs (left), tumor weight (middle) and tumor growth curve (right).

To demonstrate the role of NUPR1 in the growth and migration of liver cancer cells, we overexpressed NUPR1 in MHCC-97H and SK-Hep1 cell lines and stably knocked down NUPR1 in Huh7 and SMMC-7721 cell lines based on their expression of endogenous NUPR1(Supplementary Fig. 1A) [14]. The efficiency of these NUPR1 overexpression vectors (LV-NUPR1) and shRNAs was first assessed by qRT-PCR (Fig. 2A). Then western blot was performed to confirm the stable overexpression of NUPR1 in MHCC-97H and SK-Hep1 cells and downregulation in Huh7 and SMMC-7721 cells, respectively (Fig. 2B and Supplementary Fig. 1B,C). Subsequently, CCK-8, EdU and colony formation assays were performed to explore the effect of NUPR1 on HCC cell proliferation. As shown in Fig. 2C, NUPR1 overexpressing HCC cells exhibited a significantly enhanced proliferation rate. Similarly, in the EdU assay, NUPR1 upregulation increased the percentage of EdU‐positive cells (Fig. 2D). Moreover, downregulating NUPR1 expression significantly inhibited the proliferation of Huh7 and SMMC-7721 cells leading to fewer colonies (Fig. 2C, G). MHCC-97H cells with LV‐NC/LV-NUPR1 and SMMC-7721 cells with blank/shRNA1 were injected subcutaneously into BALB/c naked mice. NUPR1 overexpression significantly increased tumor volume and weight in the xenograft mouse model (Fig. 2E), while NUPR1 knockdown suppressed tumor growth (Fig. 2H). NUPR1 overexpression in xenograft tumor samples was further demonstrated by IHC (Fig. 2F). Overall, these findings suggest that NUPR1 can promote the proliferation of HCC cells and tumor growth in vitro and in vivo.

### NUPR1 facilitates the migration of HCC cells

**Fig. 3:**
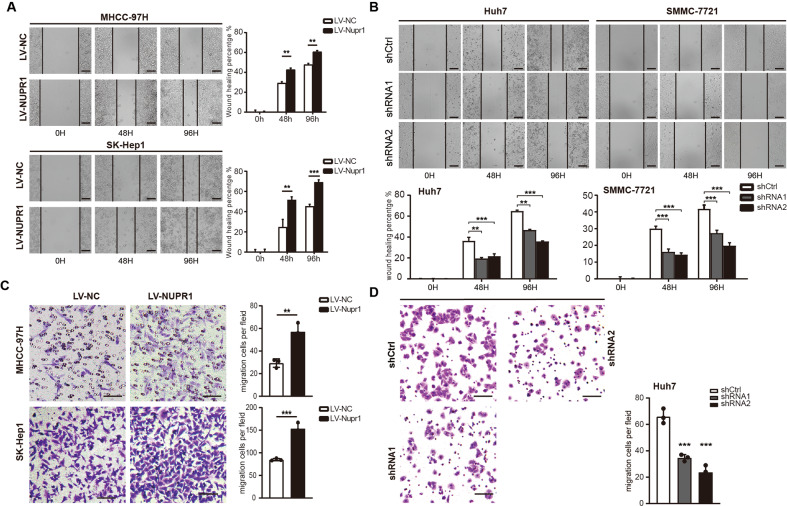
Detection of the migrative capability of HCC cells infected by NUPR1. **A** Migrative ability of MHCC-97H and SK-Hep1 cells overexpressed NUPR1 was analyzed by wound healing assays. **B** Migrative ability of Huh7 and SMMC-7721 transfected with sh-NUPR1 was analyzed using wound healing assays. **C** Migrative ability of MHCC-97H and SK-Hep1 cells transfected with LV-NUPR1 was analyzed by transwell migration assays. **D** Migrative ability of Huh7 transfected with sh-NUPR1 was analyzed using transwell migration assay.

The wound healing and transwell migration assays were performed to evaluate the effects of NUPR1 on cell migration. The cell scratch test revealed that NUPR1 overexpression significantly enhanced the wound gap closure in MHCC-97H and SK-Hep1 cells compared to the control group (Fig. 3A). Knockdown of NUPR1 significantly suppressed these changes in Huh7 and SMMC-7721 cells (Fig. 3B). Meanwhile, in the transwell migration assay, NUPR1 overexpressing tumor cells (MHCC-97H_LV-NUPR1_, 57.00 ± 4.359, and SK-Hep1_LV-NUPR1_, 153.3 ± 7.126) exhibited a significantly more invasive phenotype than control cells (MHCC-97H_LV-NC_, 29.33 ± 2.186, and SK-Hep1_LV-NC_, 85.00 ± 1.528) (*p* < 0.01) (Fig. 3C). However, NUPR1 knockdown suppressed the migration ability of Huh7 cells (Fig. 3D). These findings suggest that NUPR1 has a stimulatory effect on cell migration in HCC cells.

### NUPR1 interacts with mSREBP1 and induces nuclear entry of mSREBP1

**Fig. 4:**
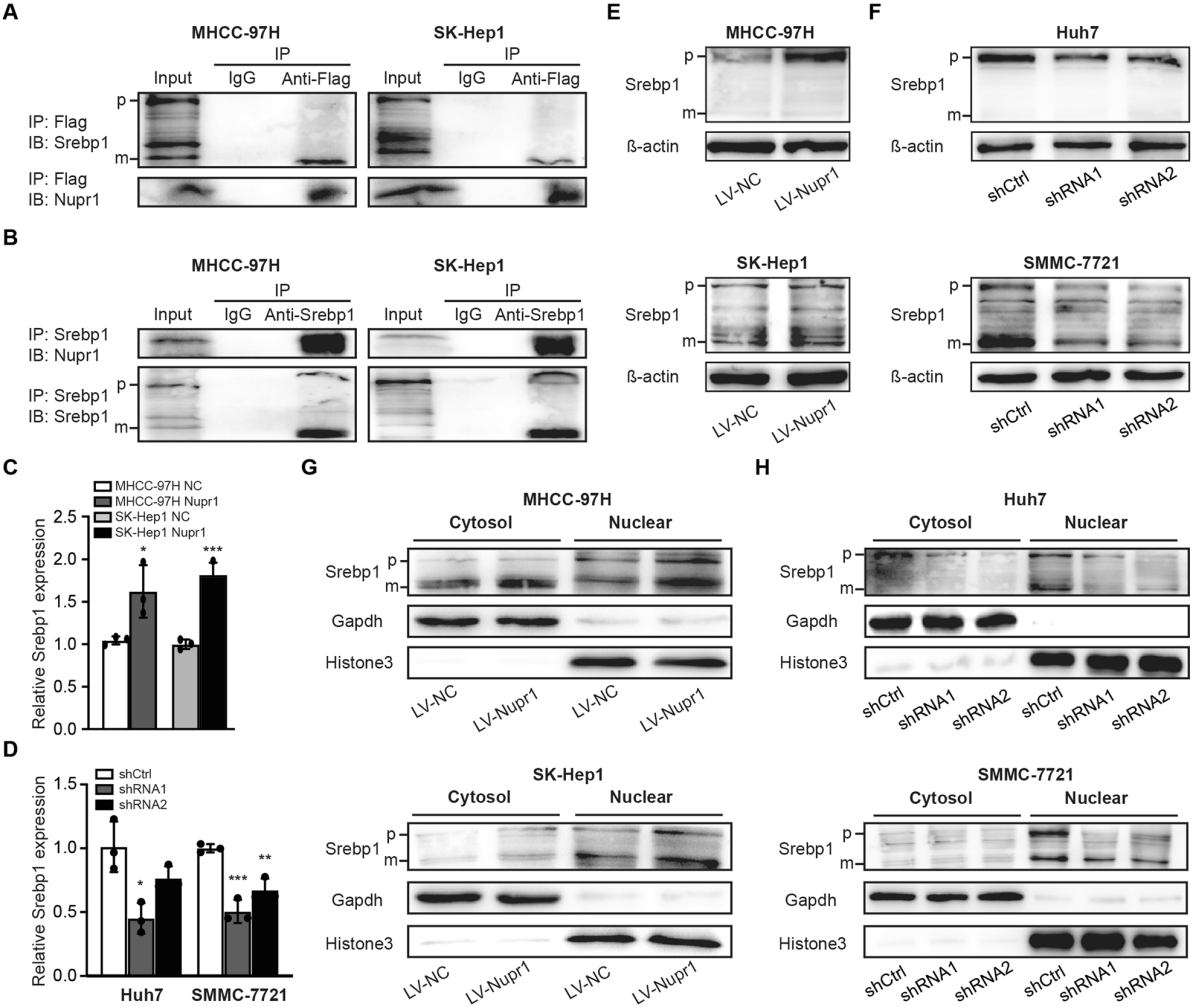
NUPR1 interacts with SREBP1 and regulates SREBP1 expression and nuclear entry in HCC cells. **A**, **B** Co-immunoprecipitation assay was conducted to detect the interaction between Flag-NUPR1 and SREBP1proteins in MHCC-97H and SK-Hep1 cells. Cell lysates were immunoprecipitated with anti-Flag antibody (a) or anti-Srebp1 antibody (b), and then the precipitates were detected with anti-Srebp1 antibody or anti-Nupr1 antibody. **C**, **D** The relationship between NUPR1 and SREBP1 was tested by RT-qPCR. **E**, **F** The relationship between NUPR1 and SREBP1 was tested by western bolt. **G**, **H** HCC cells were transfected as indicated, cells were lysed for immunoblotting of the cytosolic and nuclear SREBP1, the filters were then blotted with anti-Gapdh or anti-Histone3 as loading controls.

The above experimental results suggested that NUPR1 was associated with a proliferative and aggressive phenotype in HCC cells. To further explore the underlying molecular mechanisms, we collected human RNA-seq data from TCGA database LIHC project. Differentially expressed upregulated genes between the high NUPR1 expression and low expression groups were subjected to KEGG pathway enrichment analysis. We found a strong association between NUPR1 and non-alcoholic fatty liver disease (hsa04932) as well as cholesterol metabolism (hsa04979) (Supplementary Table 1) [15]. It has been established that transcriptional coregulators can physically interact with transcription factors and regulate a common subset of target genes of these transcription factors [16]. Accordingly, we sought to identify direct binding partners of NUPR1 in MHCC-97H and SK-Hep1 cells transfected with Flag-tagged NUPR1 by co-immunoprecipitation. Importantly, we identified mSREBP1 as a NUPR1-interacting protein. Besides, additional co-regulatory transcription factors are required in all SREBP-regulated promoters studied to date. As shown in Fig. 4A, we performed anti-Flag immunoprecipitation followed by immunoblotting with anti-SREBP1 and detected a band of the active mature form of SREBP1 (mSREBP1). Immunoprecipitation of endogenous SREBP1 followed by immunoblotting for NUPR1 was conducted (Fig. 4B). The above immunoprecipitation assays demonstrated the interaction between NUPR1 and mSREBP1 in MHCC-97H and SK-Hep1 cells.

**Fig. 5:**
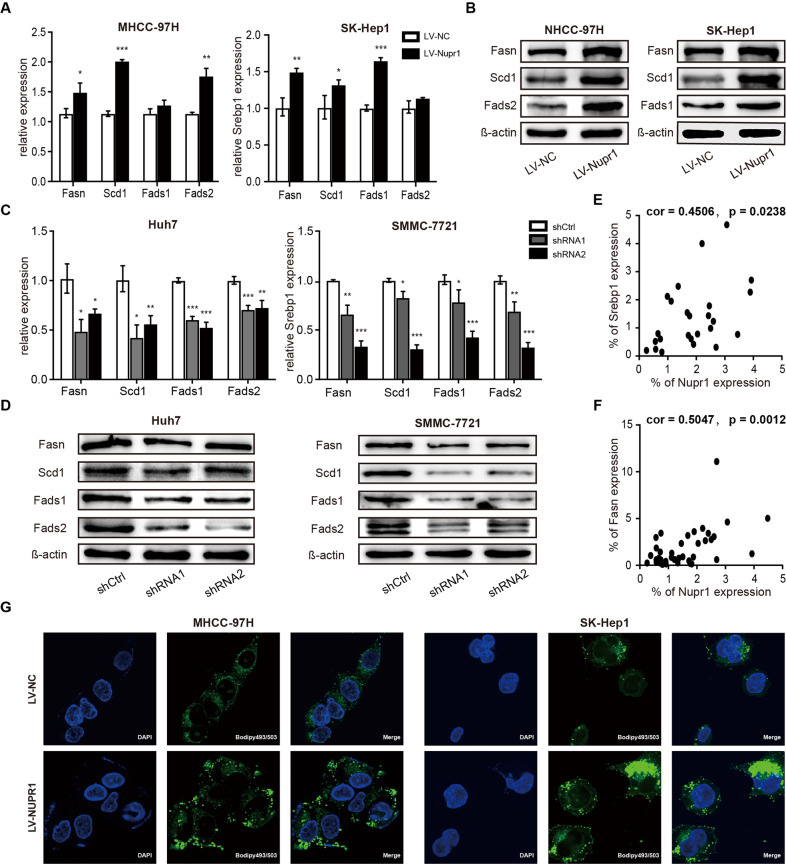
NUPR1 affected key enzyme expression of de novo lipogenesis and lipid droplet contents. **A**, **C** mRNA levels of critical lipogenesis enzymes in HCC cells with transient was detected by RT-qPCR. **B**, **D** Protein expression of critical lipogenesis enzymes in HCC cells with transient NUPR1 overexpression (b) or knockdown (d) was detected by Western blot. **E** The correlation between NUPR1 and SREBP1 was detected by immunohistochemistry in HCC patients (Pearson *R* = 0.4506, *n* = 25). **F** The correlation between NUPR1 and FASN was detected by immunohistochemistry (Pearson *R* = 0.5047, *n* = 38). **G** MHCC-97H and SK-Hep1 cells transfected with LV-NC or LV-NUPR1 stained with Bodipy 493/503 and images were acquired by confocal microscope.

Meanwhile, we analyzed the effect of NUPR1 on the expression of SREBP1 and its subcellular localization. The qRT-PCR and western blotting results showed that overexpression of NUPR1 significantly upregulated SREBP1 expression in MHCC-97H and SK-Hep1 cells compared with the control group (Fig. 4C, E), whereas downregulating NUPR1 decreased SREBP1 expression (Fig. 4D, F). Furthermore, cell fractionation and western blotting demonstrated that overexpression of NUPR1 increased the nuclear expression of SREBP1 in MHCC-97H and SK-Hep1 cells (Fig. 4G), while knockdown of NUPR1 reduced the nuclear localization of mSREBP1 (Fig. 4H). In addition, we also observed a connection between NUPR1 and SREBP1 in HCC patient samples by immunohistochemistry (IHC)(cor = 0.4506, *p* = 0.0233, Fig. 5E). Taken together, these experiments demonstrated that NUPR1 increased SREBP1 expression and nuclear localization and could bind to mSREBP1, suggesting that NUPR1 is a transcriptional coregulator of SREBP1.

### NUPR1 upregulated lipogenesis enzymes and lipid content in HCC cells

Increased nuclear SREBP1 protein has been associated with elevated mRNA levels of known SREBP1 target genes involved in fatty acid biosynthesis. Therefore, we assessed the expression of fatty acid-metabolizing enzymes in our cell lines. As shown in Fig. 5A, the mRNA expression levels of FASN, SCD1 and fatty acid desaturase 2 (FADS2) were significantly increased in NUPR1 overexpressing MHCC-97H cells, and the transcriptional levels of FASN, SCD1 and fatty acid desaturase 1 (FADS1) were upregulated in NUPR1 overexpressing SK-Hep1 cells, which were subsequently confirmed by western blot (Fig. 5B). Moreover, NUPR1 knockdown led to significant downregulation of mRNA and protein levels of FASN, SCD1, FADS1 and FADS2, compared to non-targeting shRNA control in Huh7 and SMMC-7721 cells (Fig. 5C, D). Furthermore, results from IHC of HCC patient samples showed a positive and significant correlation between NUPR1 and FASN (cor = 0.5047, *p* = 0.0012, Fig. 5F). Besides upregulation of SREBP1 and its target genes, NUPR1 overexpression also induced the accumulation of lipid droplets in HCC cells (Fig. 5G). These results indicate that NUPR1 might act as a transcriptional coregulator of SREBP1, thus elevating the expression of related target genes, leading to the accumulation of intracellular lipid droplets.

### NUPR1 promotes malignancy of HCC cells through enhancing SREBP1/FASN-mediated de novo Lipogenesis

**Fig. 6:**
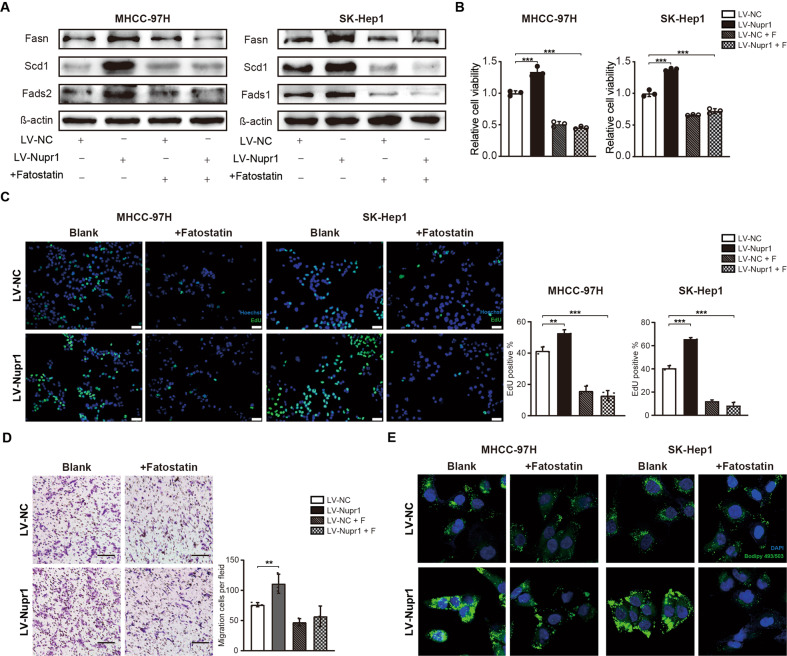
Inhibition of SREBP1 reversed the tumor-promoting effects of NUPR1 in HCC cells. **A** MHCC-97H and SK-Hep1 cells with NUPR1 overexpression were pretreated with 20 μM fatostatin for 48 h prior to collecting cell lysates. The protein expression of lipogenesis enzymes was analyzed by western blotting. **B** Effects of LV-NUPR1, fatostatin or co-transfected with LV-NUPR1 and fatostatin on the proliferation of MHCC-97H and SK-Hep1 cells were evaluated by CCK8 assays. **C** Images of MHCC-97H and SK-Hep1 transfected with LV-NC and LV-NUPR1 treated with fatostatin and the effects on the proliferation were evaluated by EdU assays, scale bars represent 20 µm. **D** Transwell migration assays were used to detecte the migrative ability of MHCC-97H cells with LV-NUPR1, fatostatin or co-transfected with LV-NUPR1 and fatostatin, scale bars represent 50 µm. **E** HCC cells transfected with LV-NUPR1, fatostatin or co-transfected with LV-NUPR1 and fatostatin were stained with Bodipy 493/503 and images were acquired by confocal microscope.

Fatostatin, a specific inhibitor of SREBP activation, has been reported to possess antitumor activity in multiple cancer types [17]. As expected, NUPR1 overexpression‐induced lipogenic enzymes expression could be suppressed by co-incubation with 20 μM Fatostatin in MHCC-97H and SK-Hep1 cells (Fig. 6A). Moreover, the fast cell proliferation rate induced by NUPR1 overexpression could also be reversed by pharmacological inhibition of SREBP1 (Fig. 6B, C). The migration ability of NUPR1-overexpressing MHCC-97H cells treated with Fatostatin was also reduced compared with the control group (Fig. 6D). In addition, HCC cells treated with Fatostatin exhibited decreased lipid droplet accumulation (Fig. 6E). Overall, these results confirmed that NUPR1 overexpression-induced cell proliferation, migration and lipid droplet accumulation could be reversed by SREBP1 inhibition in vitro.

**Fig. 7:**
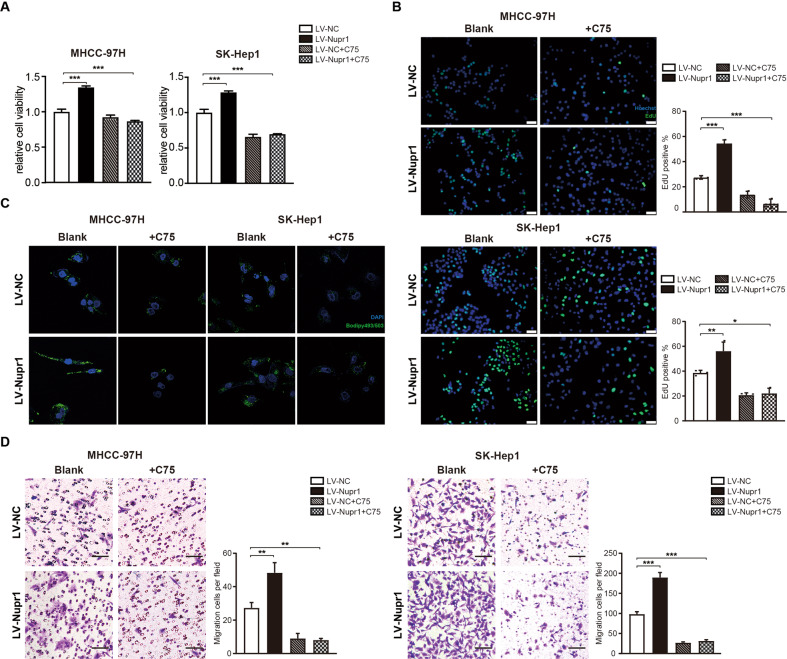
Inhibition of FASN reversed the tumor-promoting effects of NUPR1 in HCC cells. **A**, **B** Effects of LV-NUPR1, FASN inhibitor C75 (50 μM) or co-transfected with LV-NUPR1 and C75 on the proliferation of MHCC-97H and SK-Hep1 cells were evaluated by CCK8 assays (a) and EdU assays, scale bars represent 20 µm (b). **C** HCC cells transfected with LV-NUPR1, C75 or co-transfected with LV-NUPR1 and C75 were stained with Bodipy 493/503 and images were acquired by confocal microscope. **D** Transwell migration assays were used to detecte the migrative ability of MHCC-97H and SK-Hep1 cells with LV-NUPR1, C75 or co-transfected with LV-NUPR1 and C75, scale bars represent 25 µm.

The above experiments showed the effects of NUPR1 on modulating the malignant phenotype in HCC cells were dependent on SREBP1 expression. Since FASN is a key target gene of SREBP1, we hypothesized that inhibition of FASN could also reverse NUPR1-induced changes. C75, an irreversible inhibitor of FASN, was used to further examine the role of FASN in NUPR1-mediated malignant phenotype in HCC cells followed by CCK8 and transwell migration assays. As expected, NUPR1 overexpression-induced cellular proliferation could be reversed by administration of 50 μM C75 (Fig. 7A, B). Similarly, migration experiments revealed a reduced capacity for migration upon C75 treatment (Fig. 7D). As shown in Fig. 7C, lipid droplet accumulation was also reduced in HCC cells after inhibition with FASN. These observations implied that NUPR1 promoted the proliferation and migration of HCC cells by upregulating the transcriptionally active form of SREBP1, co-regulating FASN expression and promoting lipid accumulation.

## Discussion

NUPR1 is a stress-inducible nuclear protein that responds to cellular stress and features cancer initiation and development properties. The present study demonstrated that NUPR1 promoted HCC cells growth and migration contributing to hepatocellular cancer progression, consistent with the published literature. Elevated expression of NUPR1 in the context of a high-fat diet has previously been reported. Recent studies suggest NUPR1 protects tissues from cell injury in the context of obesity and high-fat diet [13]. Taken together, these findings strongly support a relationship between NUPR1 and lipid metabolism. Given that NUPR1 is highly expressed following a high-fat diet and participates in HCC pathogenesis, we sought to shed light on a possible relationship between NUPR1 and lipid metabolism in HCC.

In the present study, a novel role of NUPR1 was uncovered in LIHC progression. NUPR1 was shown overexpressed in liver tumor tissues and correlated with a poor prognosis in LIHC patients. Interestingly, we found an interplay between NUPR1 and SREBP1. Given that NUPR1 belongs to the HMG family of chromatin remodelers with transcriptional cofactor activity, it may play an important role as a transcriptional coregulator. In subsequent experiments, we found that NUPR1 promoted HCC cell proliferation and migration by facilitating nuclear translocation of the transcriptionally active form of SREBP1 and transactivating target genes such as FASN to promote lipogenesis. Pharmacological or genetic blockade of the NUPR1-SREBP1/FASN pathway enhanced anticancer activity in vitro and in vivo. These results indicate that NUPR1 plays a cancer-promoting role by enhancing SREBP1-mediated expression of FASN and de novo lipogenesis in HCC cells. While NUPR1 has been reported in the initiation and progression of tumors, little is known on the mechanism(s) underlying the regulation of lipid metabolism of cancer cells by NUPR1.

It is widely acknowledged that actively proliferating cells, especially tumor cells, have increased demands for lipids and are dependent on de novo synthesis. An enhanced de novo lipogenesis in cancer cells has long been recognized as an important characteristic of malignant tumors [18]. In this regard, elevated expression of SREBP1 has been observed in numerous types of cancer and linked to aggressive and malignant phenotypes [19, 20]. What’s more, additional co-regulatory transcription factors are required for SREBP-regulated promoters. An increasing body of evidence suggests that the maturation and nuclear translocation of SREBP1 are regulated by a variety of proteins, protein-protein interactions and epigenetic modification, which elegantly link the extracellular signals, such as insulin, or intracellular signals, such as oxidative stress, to lipid biosynthesis by modulating the transcriptional activity of SREBP1 [21, 22]. For highly proliferating cells, tumor-associated FASN is necessary for membrane lipid production and lipid droplet formation to support the increased proliferation and metabolism [23]. Unsaturated fat is a type of fatty acid with at least one double bond within the fatty acid chain that can be catalyzed by fatty acid desaturases. Unsaturated fatty acids are a key component of the phospholipids in cell membranes and help maintain membrane fluidity [24]. There is ample evidence to suggest that increased lipid unsaturation is a metabolic marker for cancer stem cells, and unsaturated fatty acids are involved in tumor progression [25, 26]. Borrello et al. pointed out that NUPR1 protected the liver from lipotoxicity by regulating fatty acid metabolism [27]. In our study, the role of NUPR1 as a regulator of fatty acid metabolism was substantiated. Although considerable efforts have been made to determine the mechanisms of lipogenic enzymes regulation, it is still unclear how dysregulation of fatty acids affects cell fate in cancer.

Previous studies have demonstrated that inhibition of NUPR1 by ZZW-115, a strong NUPR1 inhibitor, yielded a powerful anticancer effect in HCC in vitro and in vivo [28]. More importantly, we demonstrated that high NUPR1-expressing hepatocellular cancer cells showed a strong proliferative ability in vitro and in vivo, while NUPR1 knockdown inhibited tumor growth in the subcutaneous xenograft model. We also found that hepatocellular cancer cells with higher NUPR1 expression levels exhibited a more invasive phenotype. Collectively, NUPR1 is a promising therapeutic target of HCC and ZZW-115 has huge prospects for clinical application in treating liver cancers.

However, this study had several limitations. First, the underlying mechanism of NUPR1 in the lipid metabolism of HCC cells was not clarified in the present study. Moreover, it should be borne in mind that several fatty acid desaturases may be regulated by NUPR1, while the specific role of unsaturated fatty acids was not investigated. Besides, functional enrichment analysis showed NUPR1 was associated with non-alcoholic fatty liver disease, a major risk factor for HCC. However, the functional significance of NUPR1 in non-alcoholic fatty liver disease was not addressed. Another limitation in this study was that NUPR1 was expressed mainly in the areas surrounding the inflammatory infiltrates or fibroblast infiltration based on our IHC staining results. In addition, NUPR1 has been reported to play a crucial role in the fibrosis of the liver, pancreas and kidney [15, 29, 30]. There may be a close relationship between NUPR1 and the microenvironment in chronic hepatitis and liver cancer progression. Further studies are warranted to investigate the specific underlying mechanisms.

## Conclusions

In summary, our findings provide novel insights on NUPR1 function and describe a putative mechanism of increased HCC cell proliferation and migration via regulation of lipid synthesis. Importantly, NUPR1 is a highly promising therapeutic target for HCC patients.

## Materials and methods

### Bioinformatics analysis

First, we compared NUPR1 expression between tumor and normal tissues across diverse cancer types using publicly available online Tumor Immune Estimation Resource 2.0 (TIMER 2.0) database, a comprehensive resource from The Cancer Genome Atlas (TCGA). Subsequently, we explored the association between NUPR1 expression and the clinical outcome of LIHC patients using the Cox proportional hazard model, adjusted by clinical stage using TIMER 2.0. In addition, we divided the LIHC samples into three groups based on their degree of NUPR1 expression (upper quartile; interquartile range; lower quartile) and compared the 10-year overall survival between the upper quartile group (High Group) and lower quartile group (Low Group). As for enrichment analysis, we collected human LIHC RNA-seq data from TCGA database LIHC project and divided the tumor samples into three groups based on their degree of NUPR1 expression (upper quartile, High Group; interquartile range; lower quartile, Low Group). The differentially expressed upregulated genes between the High NUPR1 expression and Low expression groups were subjected to Kyoto Encyclopedia of Genes and Genomes (KEGG) pathway enrichment analysis using the R package clusterProfiler.

### Patient samples

Formalin-fixed, paraffin-embedded specimens, including primary carcinoma specimens (*n* = 50) and corresponding non-tumor normal tissues specimens used for IHC were collected from HCC patients. All clinical specimens were derived from surgeries performed at Nanfang Hospital and were confirmed as HCC by a pathologist. All experiments involving human tissues were in accordance with the principles of the Declaration of Helsinki and approved by the Institutional Review Board of Nanfang Hospital (Table 1).

**Table 1:** Clinicopathologic characteristics of hepatocellular carcinoma patients.

Characteristics	*n* = 50 (%)
Age(years)	
≤60	34 (68)
>60	16 (32)
Gender	
Male	44 (88)
Female	6 (12)
BCLC Staging	
A	4 (8)
B	5 (10)
C	40 (80)
D	1 (2)
Differentiation	
Well	5 (10)
Moderate	34 (68)
Poor	11 (22)
HBsAg	
Positive	45 (90)
Negative	5 (10)

### Immunohistochemistry assays

Tissues were fixed with 10% formalin solution and embedded in paraffin. 5 µm-thick sections were cut and baked for 60 min at 60 °C. Then, the tissue sections were deparaffinized with xylenes, rehydrated in graded ethanol, and then brought to distilled water. For antigen retrieval, sections were submerged in citrate buffer (pH 6.0) in a pressure cooker at high pressure for 5 min. Endogenous peroxide was blocked with 3% hydrogen peroxide, and 5% bovine serum albumin in PBS solution was added for 30 min to block nonspecific binding at room temperature, followed by primary antibody NUPR1 (1:150 dilution, Proteintech, Beijing, China; 15056-1-AP) incubation overnight at 4 °C. The following day, tissue sections were washed with PBS three times, incubated with secondary antibody for 60 min and washed again. After immunostaining with a DAB kit (MXB biotechnologies), sections were counterstained with hematoxylin, dehydrated and sealed.

### Cell culture and viral infection

The MHCC-97H, SK-Hep-1, Huh7 and SMMC-7721 cell lines were obtained from ATCC and cell authentication was performed via STR profiling and species authentication. Cells were cultured in Dulbecco’s modified eagle medium (DMEM) containing 10% fetal bovine serum (FBS) at 37 °C in 5% CO_2_. Cells were passaged before in vivo implantation, and adherent cells were harvested with 0.25% trypsin. Lentiviruses for overexpression of NUPR1 (LV-NUPR1) and NUPR1 shRNAs were designed and synthesized by wzbio (Shandong, China). For lentiviral transduction, the cell lines were cultured in 24-well plates containing 0.5 ml of DMEM medium supplemented with 5 mg/ml polybrene (Sigma, Shanghai, China) and 20 µl of viral concentrates. After 12 h infection, cells were washed and allowed to recover for 24 h before any further procedure.

### Western blot analysis

Total protein extracts were obtained by lysing tumor cells in RIPA lysis buffer (Beyotime, P0013B) containing phosphatase and protease inhibitors (Kangwei, CW2200S and CW2383S). Protein concentration was analyzed by the Bradford assay (Sigma-Aldrich). Samples were size-fractionated by SDS-PAGE and then transferred to polyvinyl difluoride membranes. Blots were incubated overnight at 4 °C with primary antibody, followed by exposure to the secondary antibody goat anti-rabbit or anti-mouse IgG. Nuclear and cytoplasmic fractions were separated using a Nuclear and Cytoplasmic Protein Extraction Kit (Beyotime, P0028) according to the manufacturer’s instructions.

### Co-immunoprecipitation

For protein immunoprecipitation, cells (1 × 10^7^) overexpressed Flag-tagged NUPR1 were harvested, and lysate samples containing 2000 μg total protein were immunoprecipitated with 4 μl M2-Flag (Sigma) or SREBP1 (Santa Cruz Biotechnology) respectively at 4 °C overnight, followed by incubation with 40 μl Protein A/G plus agarose beads (Santa Cruz Biotechnology) at 4 °C for 4 h. Beads were washed and boiled in 40 µl of loading buffer, and then western blotting was conducted using SREBP1 (Proteintech, 14088-1-AP) or anti-NUPR1 (Proteintech, 15056-1-AP). The protein of input used in Fig. 4-A was about 280 µg and 8 μl used for IP.

### RNA isolation, qRT-PCR

Total RNA of cultured cells was isolated using TRIzol reagent (Accurate Biotechnology, AG21102) according to the manufacturer’s protocol. Then total RNA was reverse transcribed into complementary DNA using the Evo M-MLV RT premix for qRT-PCR (Accurate Biotechnology, AG11706). For data analysis, gene expression was normalized with beta-actin and expressed as the relative expression, which was determined by the threshold cycle (C_T_) as fold change = 2^–Δ(ΔCT)^, where ΔC_T_ = C_T NUPR1_ − C_T β-actin_ and Δ(ΔCT) = ΔC_T_ tumor–ΔC_T_ normal. Primer sequences are provided in Supplemental Table S2.

### Cell proliferation assay

For cell viability detection, the Cell Counting Kit-8 (CCK8) and 5-ethynyl-2′-deoxyuridine (EdU) assays were performed according to the manufacturer’s protocol. The cells were seeded in a 96-wells plate at a density of 1 ×10^3^ cells/well and cultured in a 10% FBS DMEM medium. CCK8 reagent (Dojindo, Tokyo, Japan) was added and incubated for 2 h once a day for 6 or 7 consecutive days. EdU staining was performed using the EdU labelling kit (Ribobio, C10310-1). Cell colony formation ability was measured by plate colony formation assay. Cells (1000 or 500 cells/per well) were plated into 6-well plates and cultured for 2 weeks. Colonies were fixed using 4% paraformaldehyde, stained using 0.1% crystal violet solution and counted by Image J software. Cell viability after drug treatment was assessed in the following HCC cancer cell lines: MHCC-97H and SK-Hep1. Cells were seeded in 96-well plates (4000 cells/well) on the first day. After culture overnight, the cell medium was replaced with Fatostatin (20 μM, MCE, HY-14452) or C75 (50 μM, MCE, HY-12364) on the second day. After 48 h drug treatment, CCK8 or EdU was used to measure the cell viability, and cell viability rates were compared with the untreated group.

### Transwell migration assay

The cells were digested with trypsin, centrifuged, and washed twice with PBS. The concentration of cells was adjusted to 5 × 10^5^cells/ml in a serum-free medium. The transwell chamber with 8.0 μm (for SK-Hep1, Huh7 and SMMC-7721) or 12.0 μm (for MHCC-97H) pores was inserted into a 24-well plates, 200 μl cell suspension was added in the transwell chamber. 700 μl medium containing 20% FBS was added in the lower chamber. SK-Hep1, Huh7 and SMMC-7721 cells were cultured for 10 h and MHCC-97H cells were cultured for 72 h at 37 °C in 5% CO_2_. After incubation, the chambers were washed twice with PBS, then fixed with 4% paraformaldehyde for 30 min, stained with 0.1% crystal violet for 20 min, and washed twice with PBS again. Transwell chamber results were photographed using an inverted microscope.

### Cell scratch tests

Cells were seeded in the 6-well plate with the 10% FBS medium. When the cells were grown to 90% confluence, a scratch was made across the cell monolayer with a 10 µl pipette tip to create a uniform cell-free wound area. Debris was removed by gently washing with PBS and cells were cultured in serum-free medium. The length of the cell-free area was monitored and photographed at 0, 48, and 96 h using light microscopy. Data are expressed as a percentage of the initial length at time zero.

### Bodipy staining for microscopy

Most hepatic lipid is stored in the hepatocyte as cytosolic lipid droplets, neutral lipid depots composed of triacylglycerol and cholesteryl esters. Therefore, BODIPY 493/503 was used to detect the neutral lipid depots in MHCC-97H and SK-Hep1 cells [31]. Cells were grown in a confocal Petri dish (NEST) and incubated for 72 h. Then the cells were washed with PBS and fixed with 4% paraformaldehyde. Subsequently, the cells were incubated with 2 µM BODIPY (Glpbio) staining solution, and then washed with PBS for three times. The nuclei were stained with DAPI and the cells were observed under a confocal laser microscope.

### BALB/c-nude mice tumorigenesis experiment

We created a mouse modle of cancer induced by subcutaneous injection of MHCC-97H (LV-NC and LV-NUPR1) cells or SMMC-7721 (SMMC-7721 and SMMC-7721 shNUPR1-1) cells. Five-week-old male BALB/c nude mice were purchased form the Guangdong Medical Laboratory Animal Center, Guangdong, China. The mice were randomly assigned to two groups. A total of 5 × 10^6^ cells were suspended in 100 µl of PBS and subcutaneously injected into the right flank of BALB/c nude mice. The tumorigenesis of the nude mice was observed every 3 days. Mice were sacrificed 28 days after injection, and xenografts were dissected, photographed, weighted, fixed in neutral buffered formalin and subsequently analyzed by IHC. Animal procedures were approved by the Institutional Review Board of Nanfang Hospital.

### Statistics analysis

The statistical software GraphPad Prism was used to evaluate the data in this study. An unpaired two-tailed Student’s *t* test was used to compare two groups, and comparisons between multiple groups were analyzed by one-way ANOVA with Dunnett post hoc test. Correlation analysis was assessed using the Pearson correlation coefficient. A *p value* < 0.05 was statistically significant. Error bars in all figures represent the mean ± SD. *p < 0.05; **p < 0.01; ***p < 0.001; “ns” not significant.

## Supplementary information

Supplementary figure legneds

Supplementary tables

Supplementary Figure 1

Full and uncropped western blots

## Data Availability

Some of the original data can be obtained directly from publicly available TCGA (https://portal.gdc.cancer.gov/projects/TCGA-LIHC) and TIMER2.0 (http://timer.cistrome.org/) databases, further inquiries can be directed to the corresponding author.
