# Supplementary material for: The interplay of transcriptional coregulator NUPR1 with SREBP1 promotes hepatocellular carcinoma progression via upregulation of lipogenesis
Source: Cell Death Discov. 2022 Oct 28;8:431. doi: 10.1038/s41420-022-01213-z (PMC9616853; doi:10.1038/s41420-022-01213-z)
Supplement: Supplementary file 1 — Supplementary figure legneds [file 41420_2022_1213_MOESM1_ESM.docx]

**Supplementary Figure 1** **A**. The expression of NUPR1 was detected in SK-Hep1, Huh7, SMMC-7721 and MHCC-97H cells. **B, C.** Statistical analyses of NUPR1 expression after overexpression (b) and knockdown (c) against NUPR1 in HCC cells detected by western blot.
