# Supplementary material for: The interplay of transcriptional coregulator NUPR1 with SREBP1 promotes hepatocellular carcinoma progression via upregulation of lipogenesis
Source: Cell Death Discov. 2022 Oct 28;8:431. doi: 10.1038/s41420-022-01213-z (PMC9616853; doi:10.1038/s41420-022-01213-z)
Supplement: Supplementary file 2 — Supplementary tables [file 41420_2022_1213_MOESM2_ESM.docx]

**Supplementary Table 1. Results of KEGG enrichment.**

| ID | Description | p.adjust | qvalue | Count |
| --- | --- | --- | --- | --- |
| hsa00190 | Oxidative phosphorylation | 3.44E-17 | 3.04E-17 | 52 |
| hsa05012 | Parkinson disease | 1.06E-16 | 9.38E-17 | 75 |
| hsa05020 | Prion disease | 4.97E-16 | 4.38E-16 | 74 |
| hsa05016 | Huntington disease | 4.35E-15 | 3.84E-15 | 78 |
| hsa05022 | Pathways of neurodegeneration-multiple diseases | 5.51E-15 | 4.87E-15 | 89 |
| hsa05014 | Amyotrophic lateral sclerosis | 4.24E-14 | 3.74E-14 | 79 |
| hsa04714 | Thermogenesis | 7.00E-13 | 6.18E-13 | 61 |
| hsa05010 | Alzheimer disease | 2.58E-12 | 2.28E-12 | 86 |
| hsa05415 | Diabetic cardiomyopathy | 2.03E-10 | 1.79E-10 | 48 |
| hsa01100 | Metabolic pathways | 1.50E-09 | 1.33E-09 | 180 |
| hsa05208 | Chemical carcinogenesis - reactive oxygen species | 4.23E-09 | 3.73E-09 | 56 |
| hsa04932 | Non-alcoholic fatty liver disease | 5.33E-09 | 4.71E-09 | 46 |
| hsa03050 | Proteasome | 6.33E-09 | 5.59E-09 | 25 |
| hsa03010 | Ribosome | 2.63E-05 | 2.32E-05 | 21 |
| hsa04723 | Retrograde endocannabinoid signaling | 4.68E-05 | 4.13E-05 | 24 |
| hsa04260 | Cardiac muscle contraction | 9.75E-05 | 8.61E-05 | 18 |
| hsa04145 | Phagosome | 0.008464 | 0.007471 | 16 |
| hsa00520 | Amino sugar and nucleotide sugar metabolism | 0.014178 | 0.012515 | 12 |
| hsa01240 | Biosynthesis of cofactors | 0.014601 | 0.012888 | 25 |
| hsa05110 | Vibrio cholerae infection | 0.020698 | 0.01827 | 9 |
| hsa00983 | Drug metabolism - other enzymes | 0.024107 | 0.021279 | 10 |
| hsa04612 | Antigen processing and presentation | 0.045209 | 0.039906 | 8 |
| hsa04142 | Lysosome | 0.047319 | 0.041769 | 20 |
| hsa00480 | Glutathione metabolism | 0.047319 | 0.041769 | 9 |
| hsa00240 | Pyrimidine metabolism | 0.047319 | 0.041769 | 10 |
| hsa04146 | Peroxisome | 0.047319 | 0.041769 | 12 |
| hsa04141 | Protein processing in endoplasmic reticulum | 0.04732 | 0.04177 | 26 |
| hsa04623 | Cytosolic DNA-sensing pathway | 0.089367 | 0.078884 | 8 |
| hsa04979 | Cholesterol metabolism | 0.089367 | 0.078884 | 9 |
| hsa01200 | Carbon metabolism | 0.109038 | 0.096247 | 14 |
| hsa05017 | Spinocerebellar ataxia | 0.1481 | 0.130728 | 21 |
| hsa04370 | VEGF signaling pathway | 0.248358 | 0.219225 | 10 |
| hsa05132 | Salmonella infection | 0.263212 | 0.232337 | 25 |
| hsa04721 | Synaptic vesicle cycle | 0.277699 | 0.245125 | 8 |
| hsa05323 | Rheumatoid arthritis | 0.277699 | 0.245125 | 8 |
| hsa03020 | RNA polymerase | 0.295037 | 0.260429 | 7 |

**Supplementary Table 2. Sequence and primer information.**

| Primer |  | Sequence |
| --- | --- | --- |
| β-actin | forward: | CTCCCTGGAGAAGAGCTACGAGC |
|  | reverse: | CCAGGAAGGAAGGCTGGAAGAG |
| NUPR1 | forward: | CCATTCCTACCTCGGGCCTCTCATC |
|  | reverse: | TCTTGGTGCGACCTTTCCGGC |
| SREBP1 | forward: | CGGAACCATCTTGGCAACAGT |
|  | reverse: | CGCTTCTCAATGGCGTTGT |
| FASN | forward: | TTCTACGGCTCCACGCTCTTCC |
|  | reverse: | GAAGAGTCTTCGTCAGCCAGGA |
| SCD-F | forward: | CCTGGTTTCACTTGGAGCTGTG |
|  | reverse: | TGTGGTGAAGTTGATGTGCCAGC |
| FADS1 | forward: | CTGTCGGTCTTCAGCACCTCAA |
|  | reverse: | CTGGGTCTTTGCGGAAGCAGTT |
| FADS2 | forward: | GACCACGGCAAGAACTCAAAG |
|  | reverse: | GAGGGTAGGAATCCAGCCATT |
