# Supplementary figures and images for: The interplay of transcriptional coregulator NUPR1 with SREBP1 promotes hepatocellular carcinoma progression via upregulation of lipogenesis
Source: Cell Death Discov. 2022 Oct 28;8:431. doi: 10.1038/s41420-022-01213-z (PMC9616853; doi:10.1038/s41420-022-01213-z)

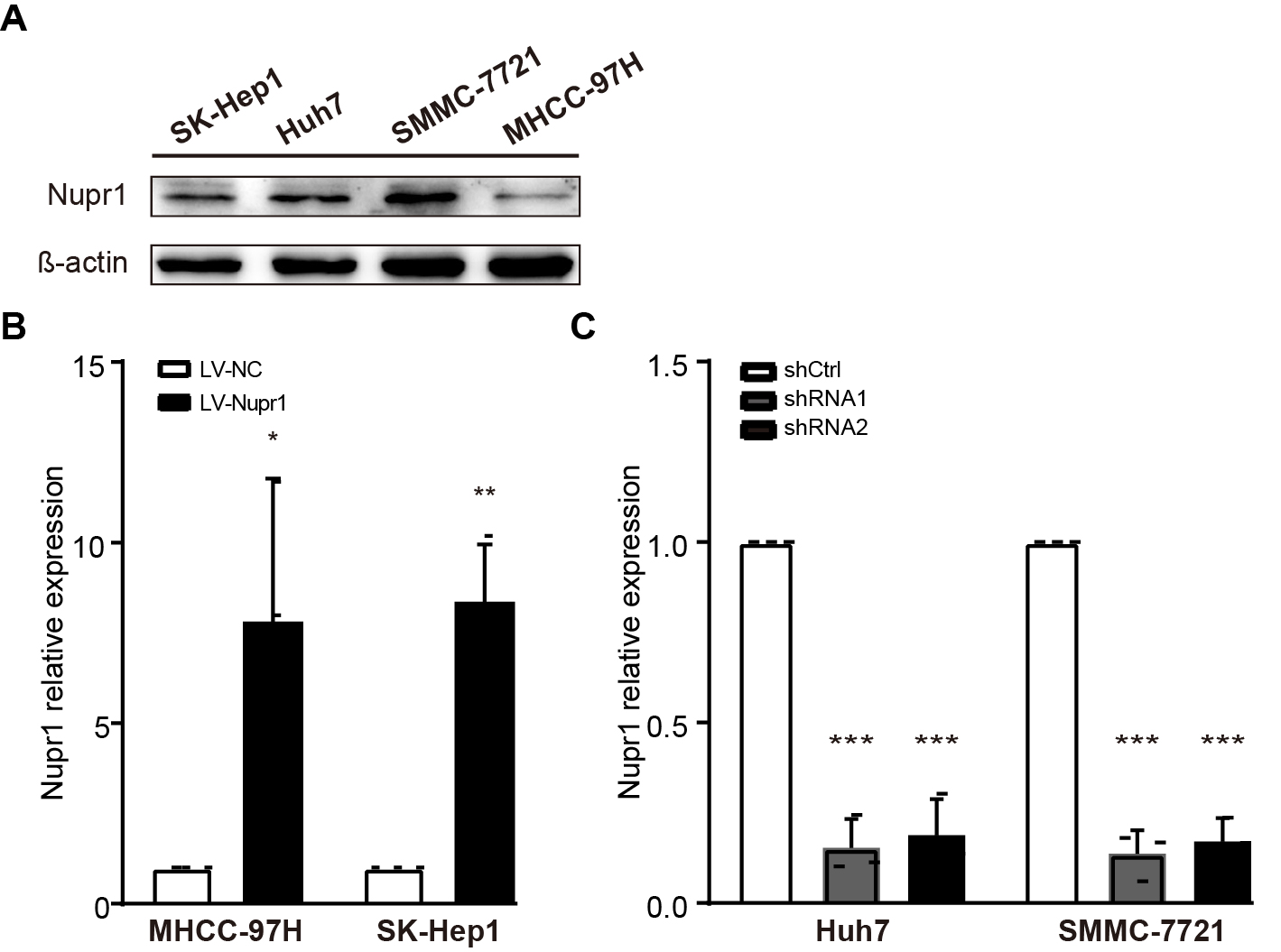

Supplement: Supplementary file 3 — Supplementary Figure 1 [file 41420_2022_1213_MOESM3_ESM.jpg]
