# Supplementary material for: The interplay of transcriptional coregulator NUPR1 with SREBP1 promotes hepatocellular carcinoma progression via upregulation of lipogenesis
Source: Cell Death Discov. 2022 Oct 28;8:431. doi: 10.1038/s41420-022-01213-z (PMC9616853; doi:10.1038/s41420-022-01213-z)

Figure 2. Huh7_ACTIN


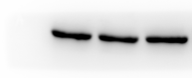


Figure2. Huh7_NUPR1


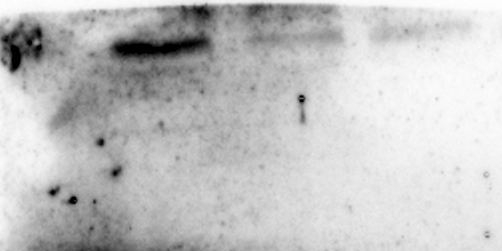


Figure2. 97H_ACTIN


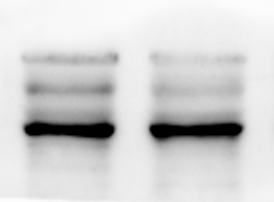


Figure2. 97H_NUPR1


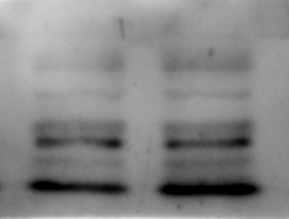


Figure2. Hep1_ACTIN


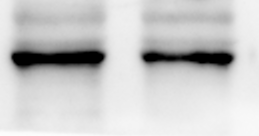


Figure2. Hep1_NUPR1


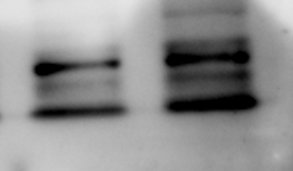


Figure2.7721_actin
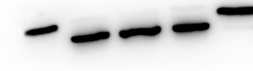


Figure2. 7721_NUPR1


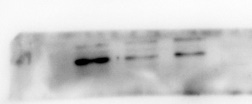


Huh7_ACTIN-2


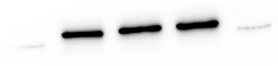


Huh7_NUPR1-2


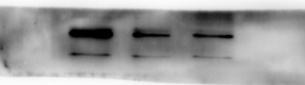


Huh7_ACTIN-3
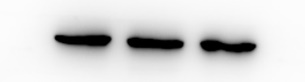


Huh7_NUPR1-3
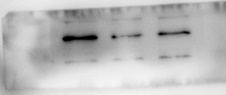


97H_ACTIN-2


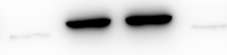


97H_NUPR1-2


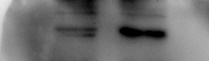


97H_ACTIN-3


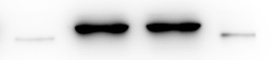


97H_NUPR1-3


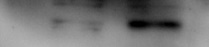


Hep1_ACTIN-2


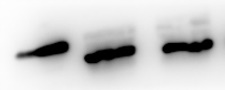


Hep1_NUPR1-2


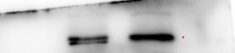


Hep1_ACTIN-3


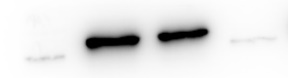


Hep1_NUPR1-3


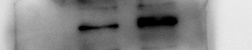


7721_actin-2


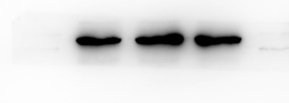


7721_NUPR1-2
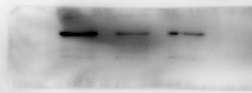


7721_actin-3


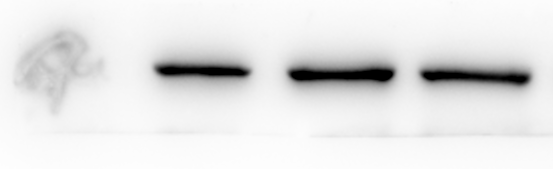


7721_NUPR1-3
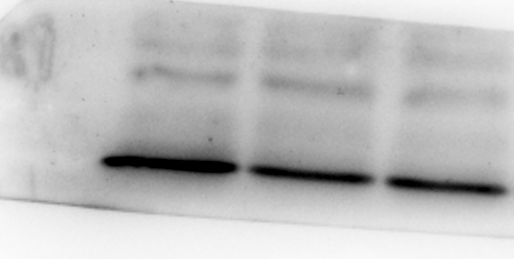


Supplementary Figure 1.

Hep1 Huh7 7721 97H_actin


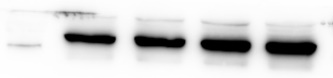


Supplementary Figure 1.

Hep1 Huh7 7721 97H_Nupr1


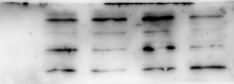


Figure 4F.Huh7_actin


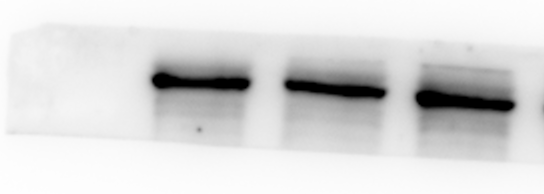


Figure 4F.Huh7_srebp1
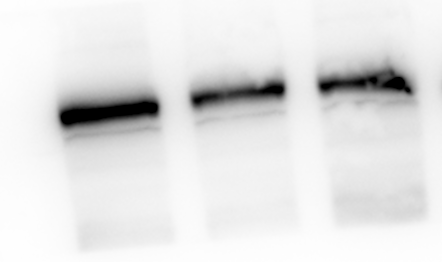


Figure 4H.Huh7_gapdh


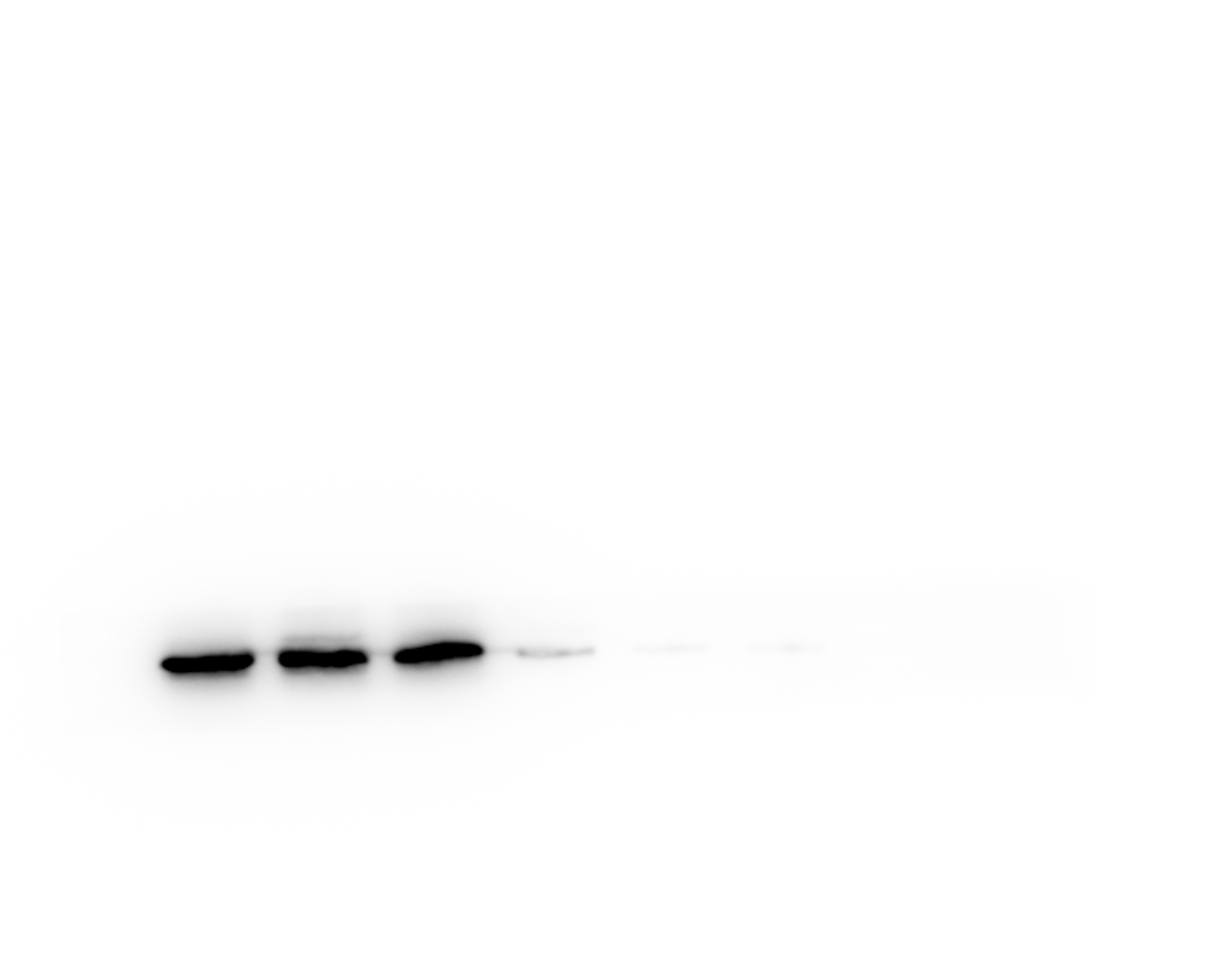


Figure 4H.Huh7_histone


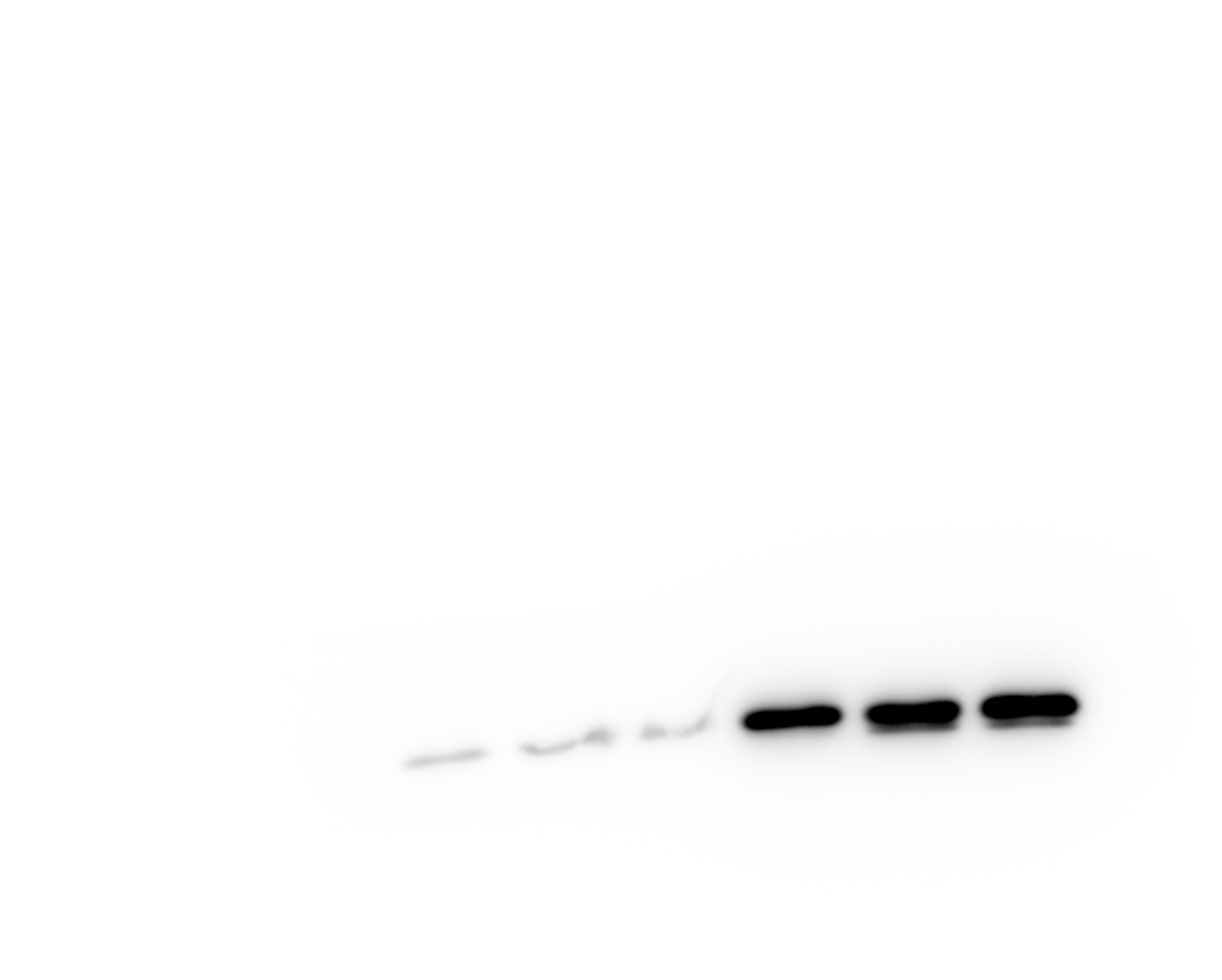


Figure 4H.Huh7_srebp1


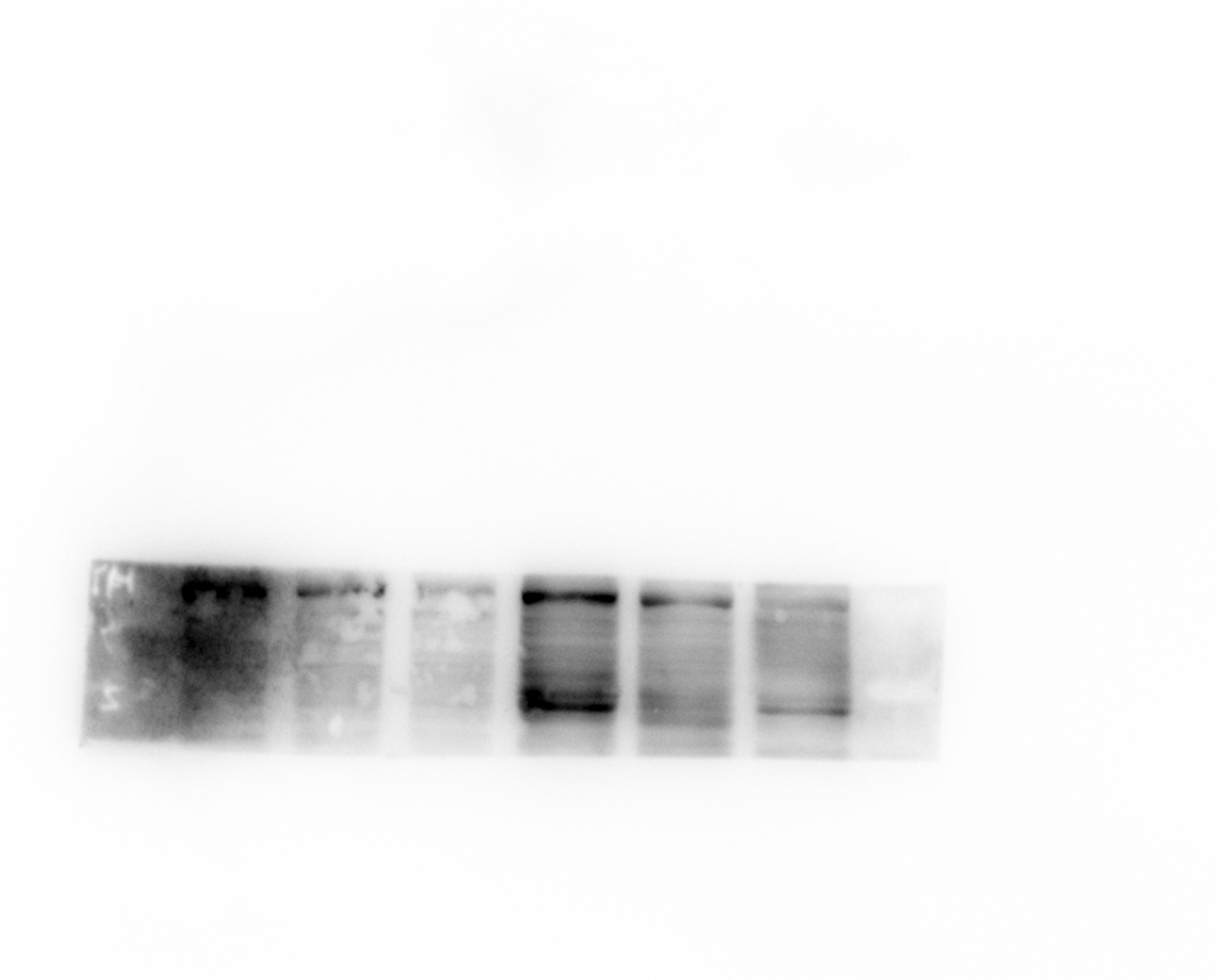


Figure 4A.97H_nupr1


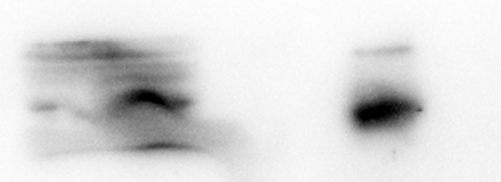


Figure 4A.97H_srebp1


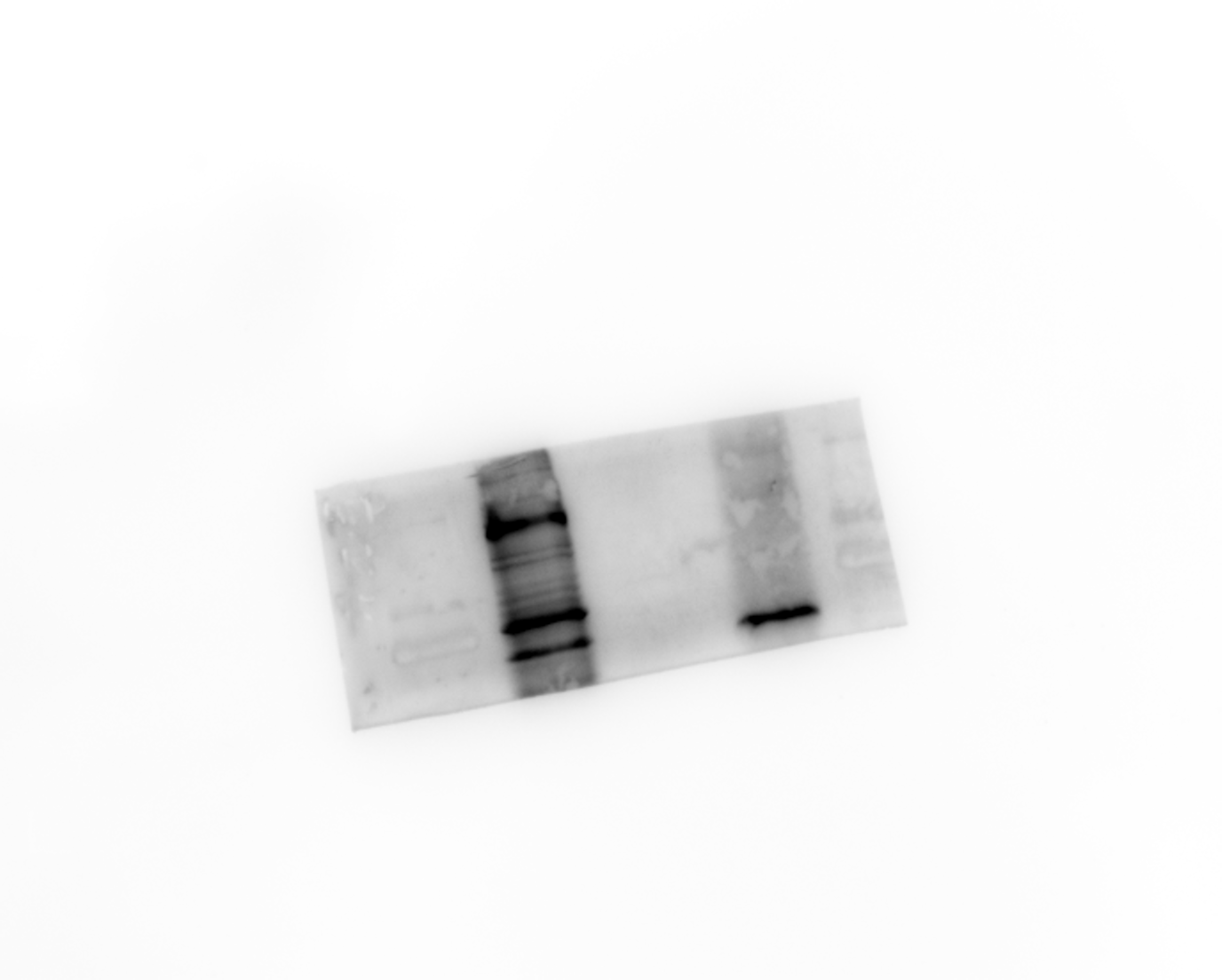


Figure 4B.97H_nupr1


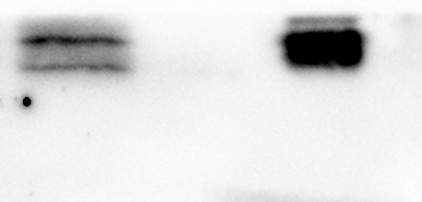


Figure 4B.97H_srebp1


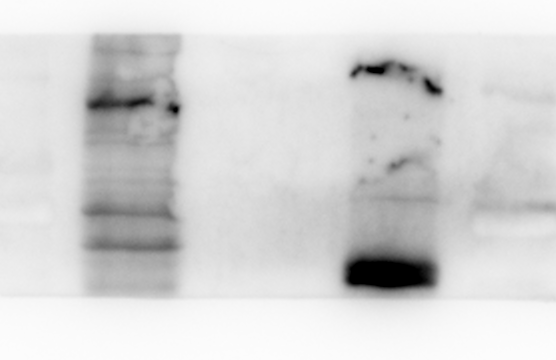


Figure 4E. 97H_actin


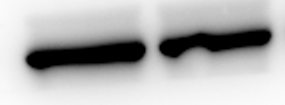


Figure 4E.97H_srebp1


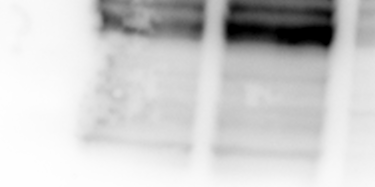


Figure 4G.97H_gapdh


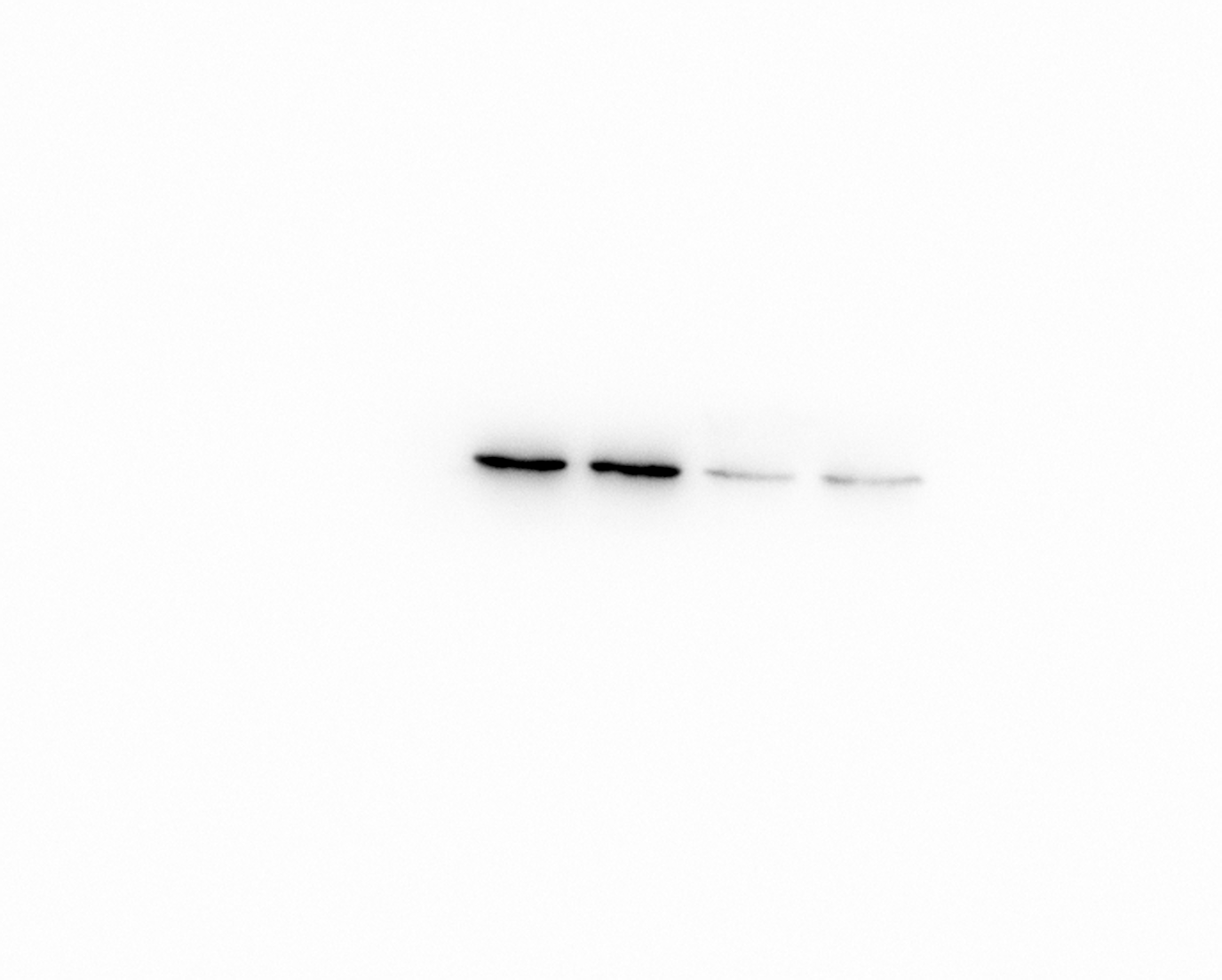


Figure 4G.97H_serbp1


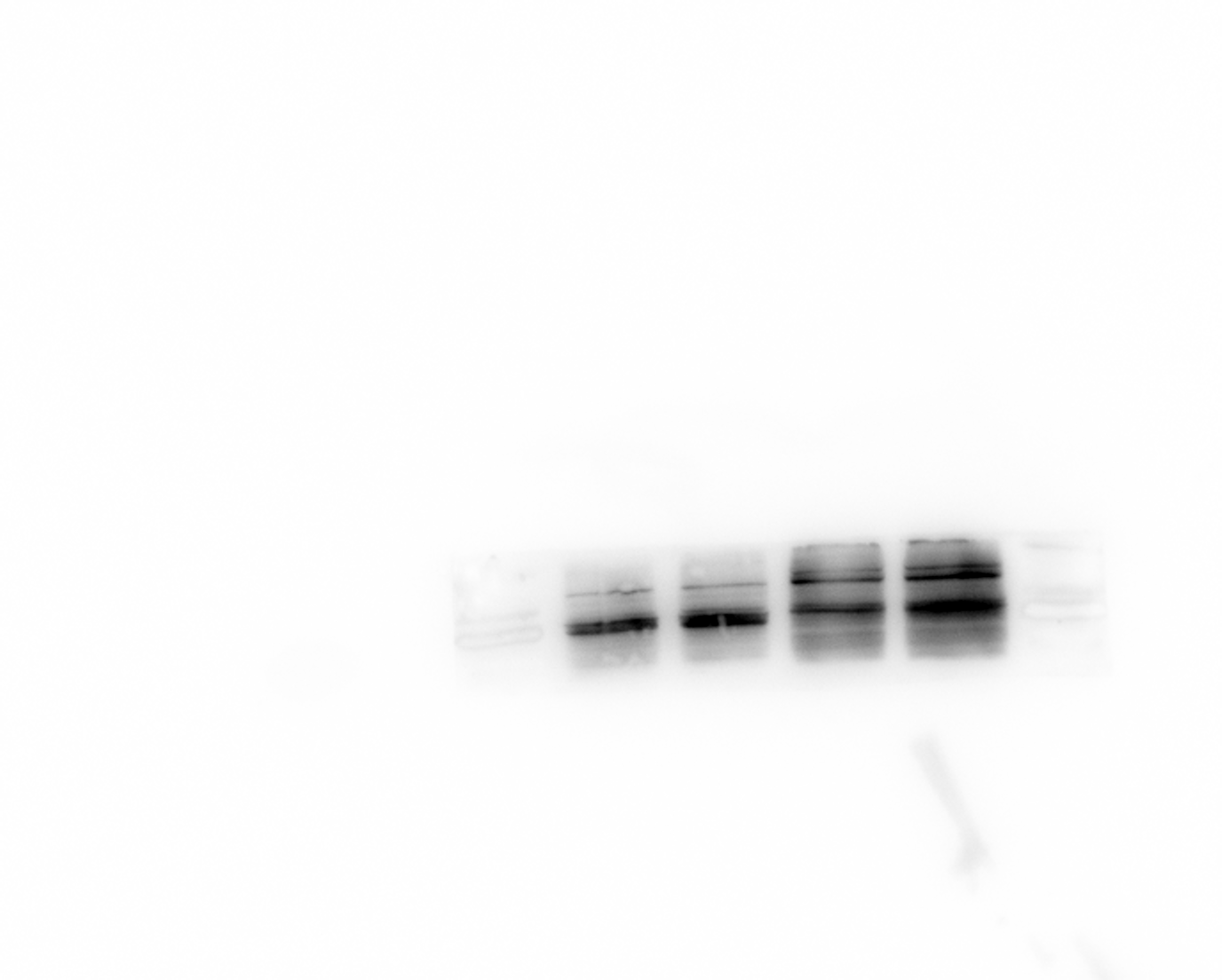


Figure 4G.97H_histone3


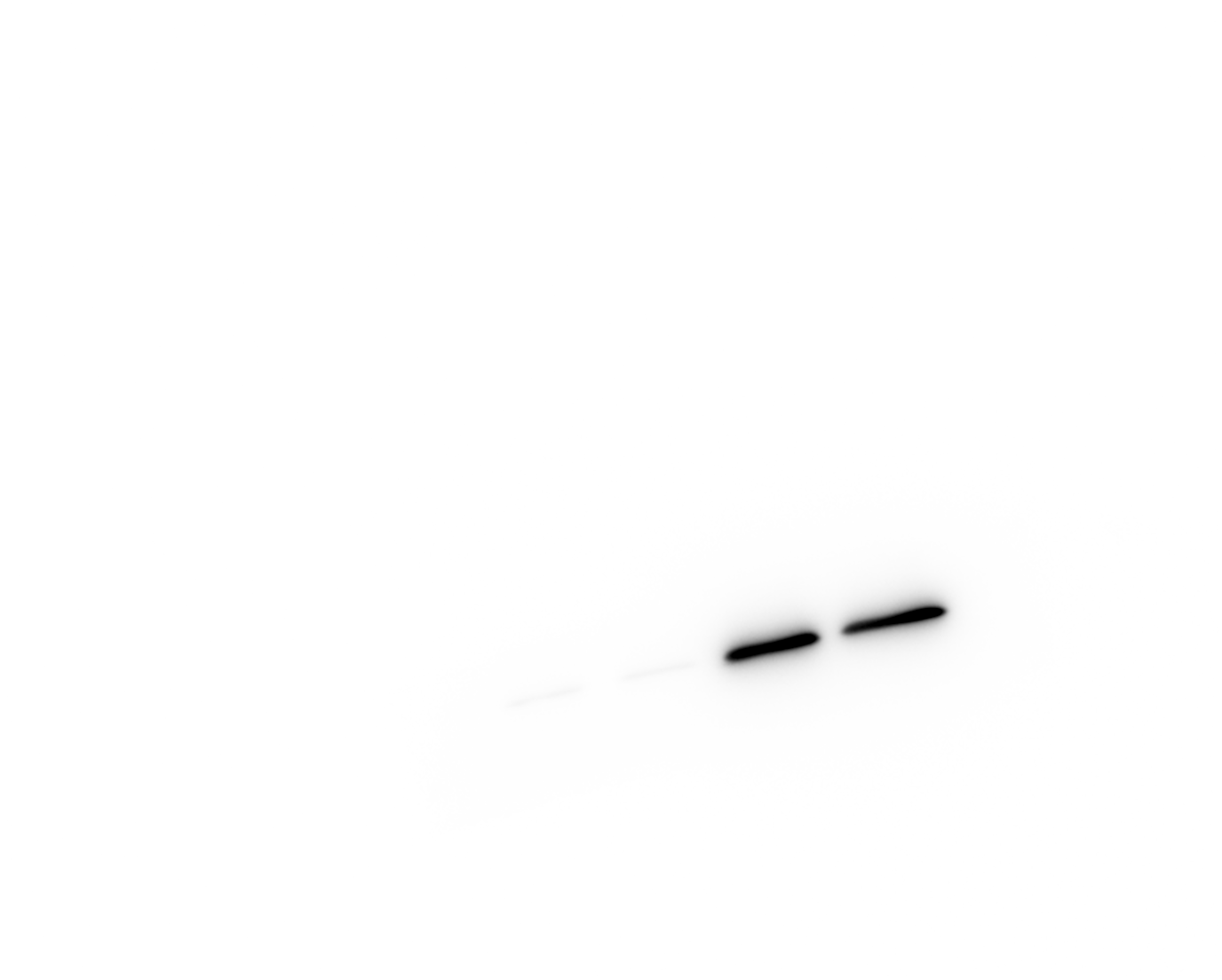


Figure 4A.Hep1_nupr1


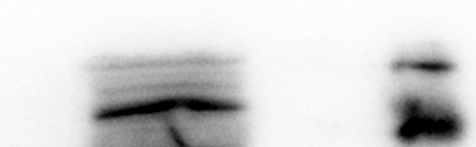


Figure 4A.Hep1_srebp1


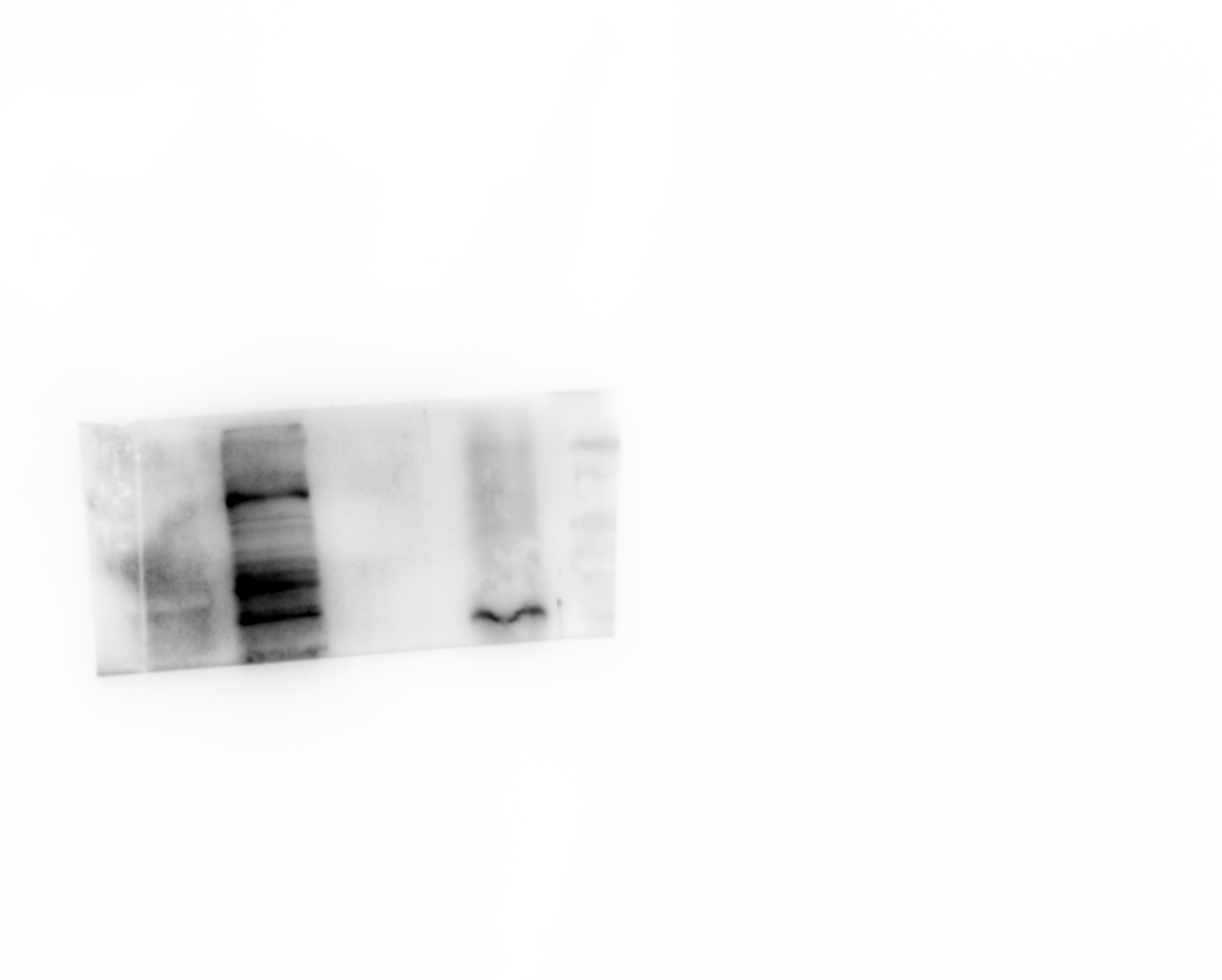


Figure 4B. Hep1_srebp1


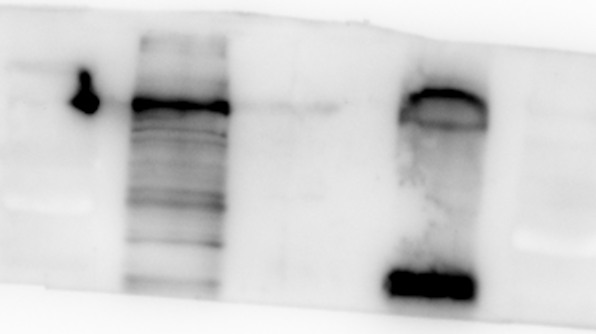


Figure 4B.Hep1_nupr1


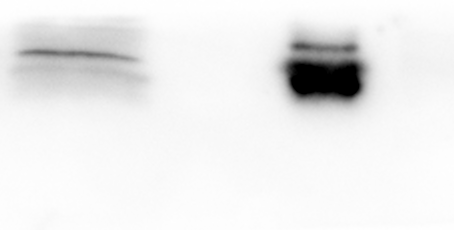


Figure 4E. Hep1_actin


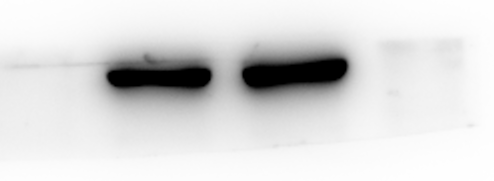


Figure 4E. Hep1_srebp1


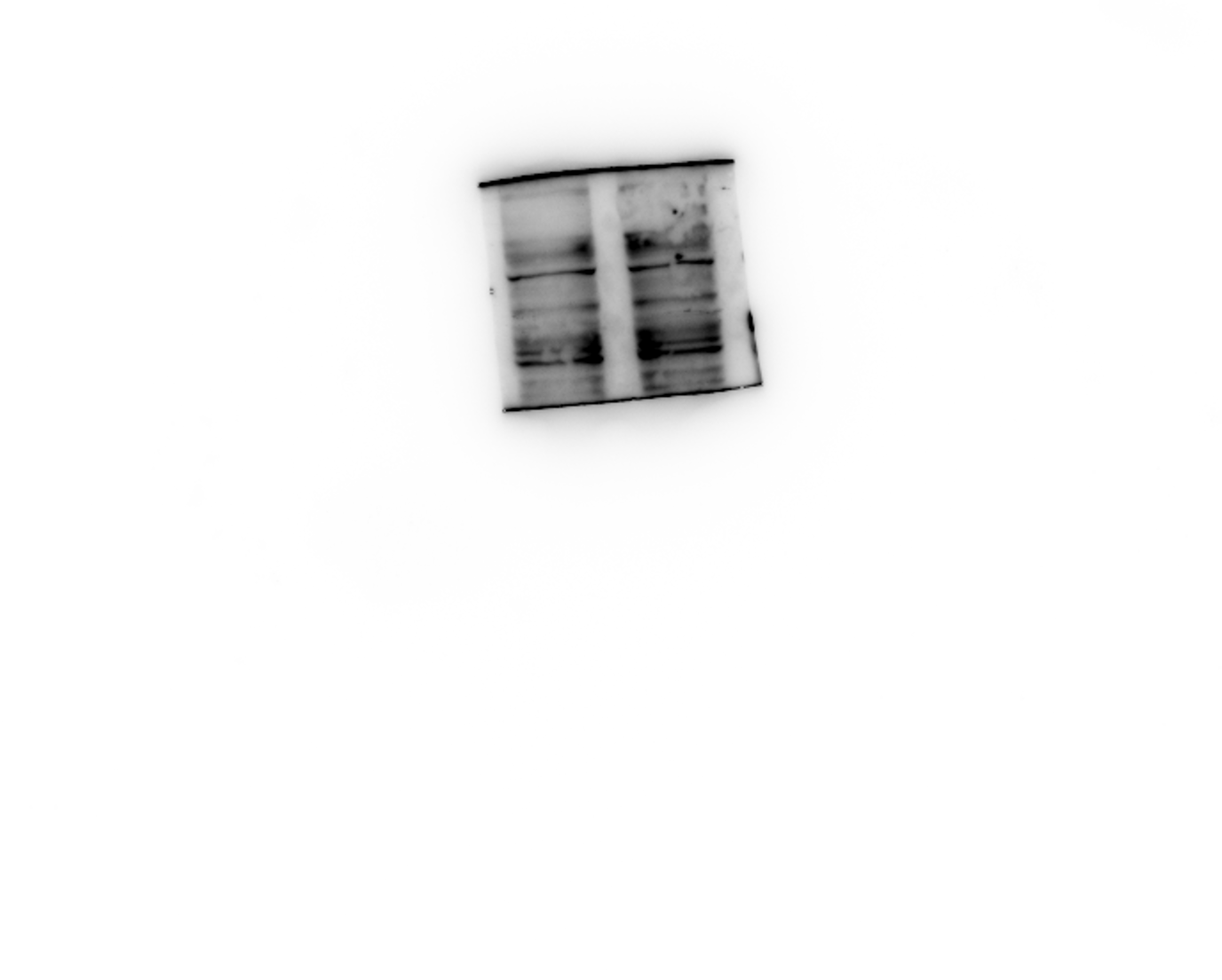


Figure 4G. Hep1_gapdh


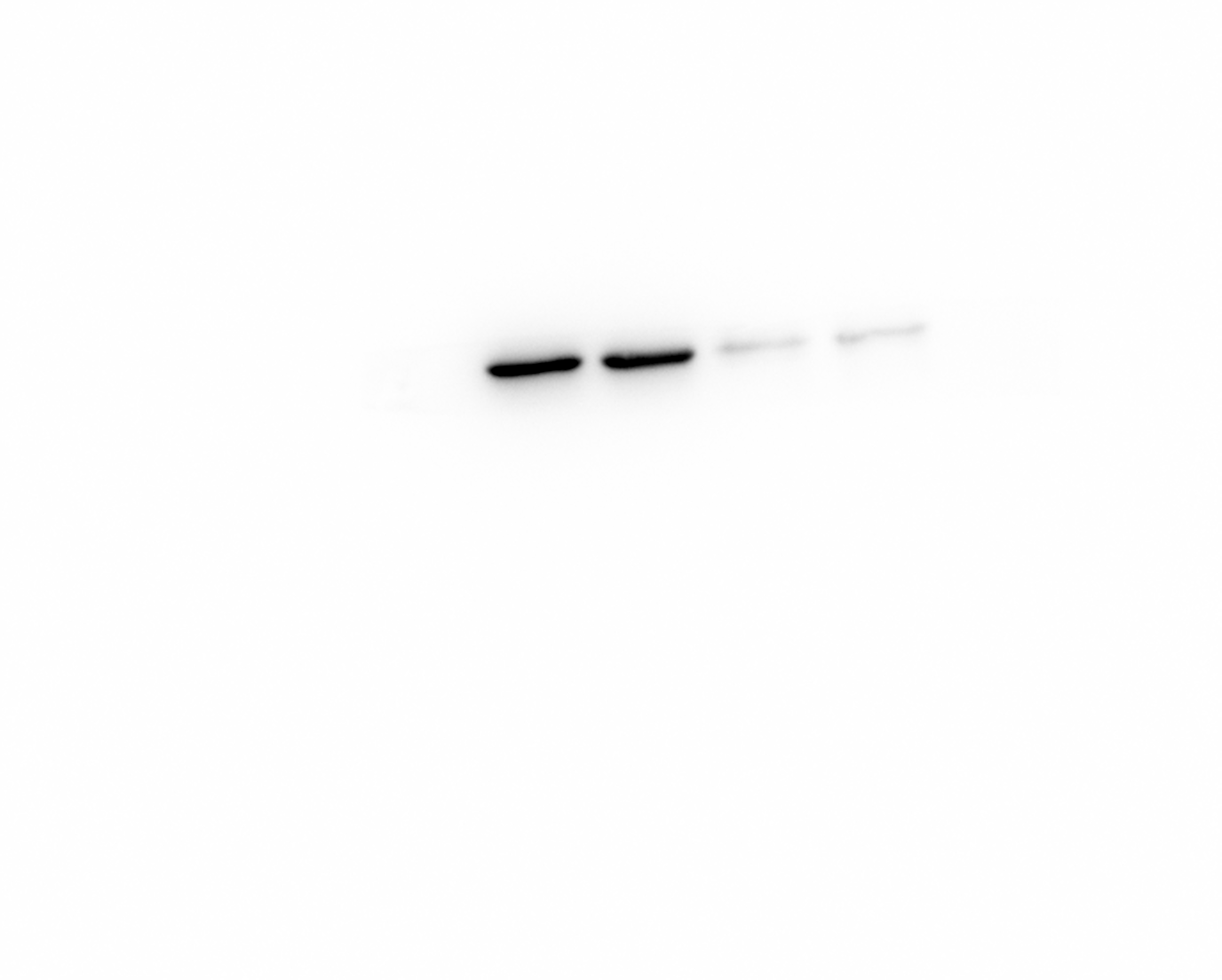


Figure 4G.Hep1_histone3


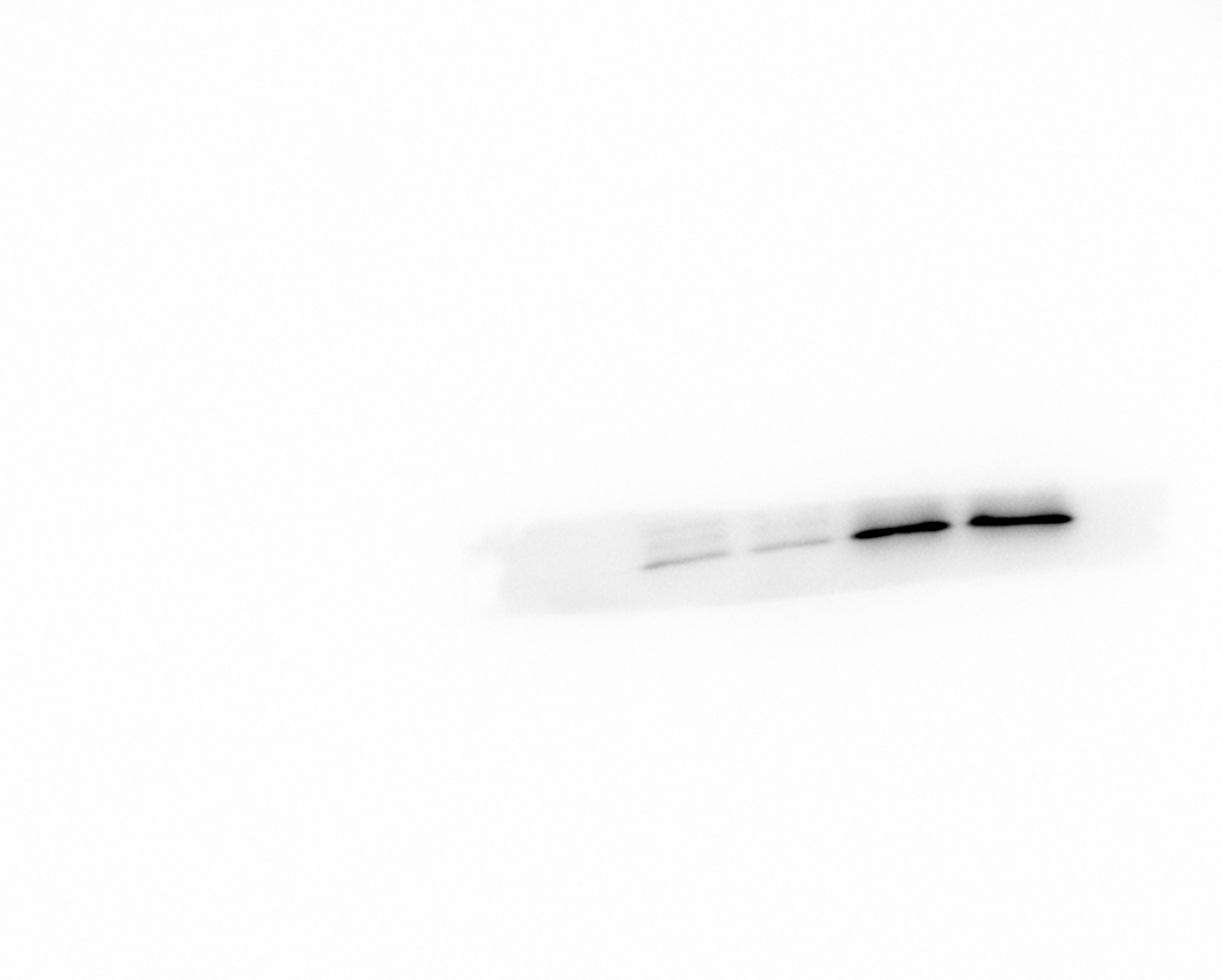


Figure 4G.Hep1_srebp1


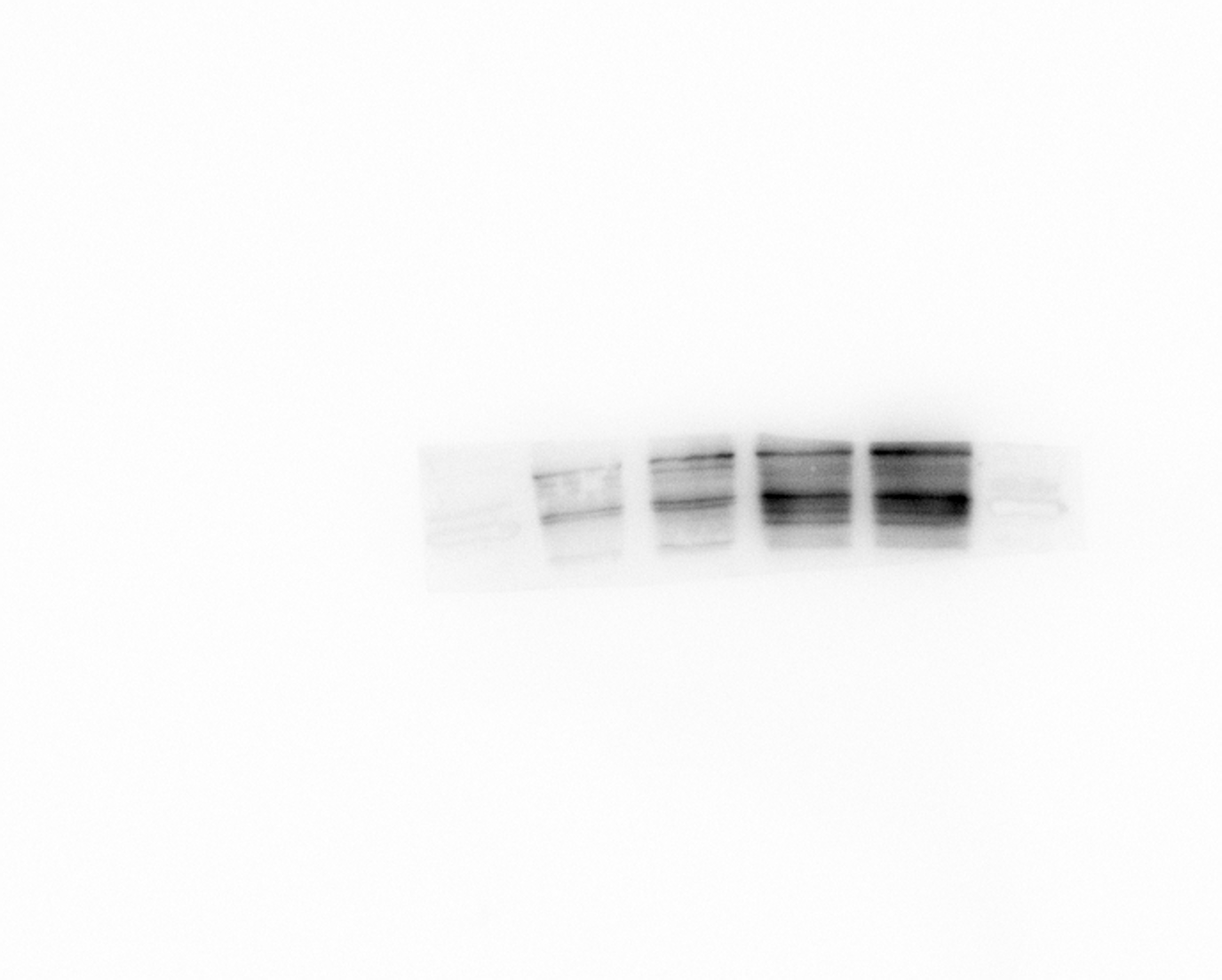


Figure 4F.7721_actin


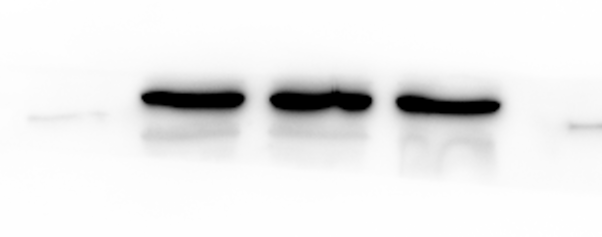


Figure 4F.7721_srebp1


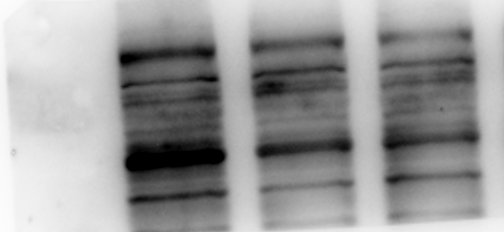


Figure 4H.7721_histone3


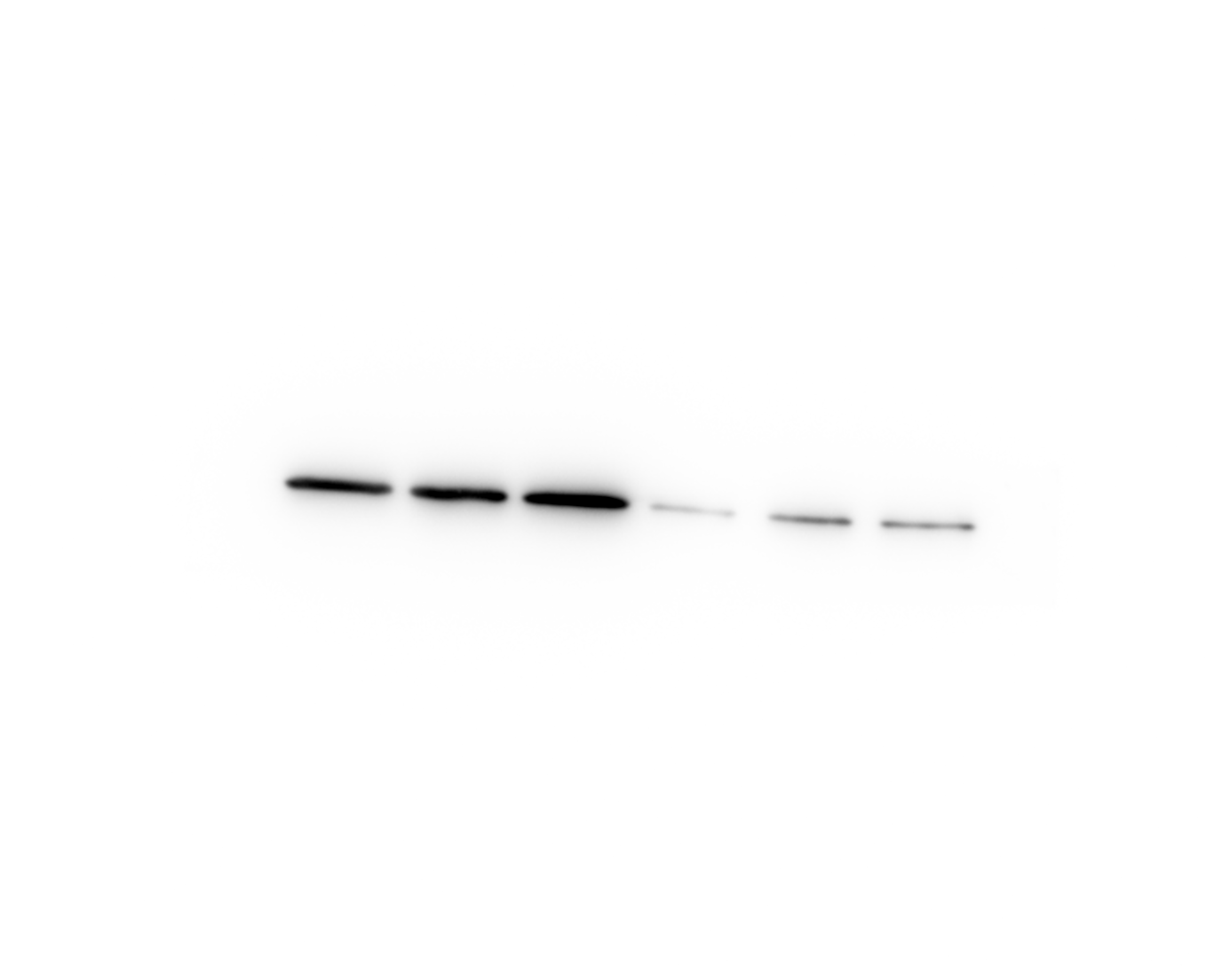


Figure 4H.7721_histone3


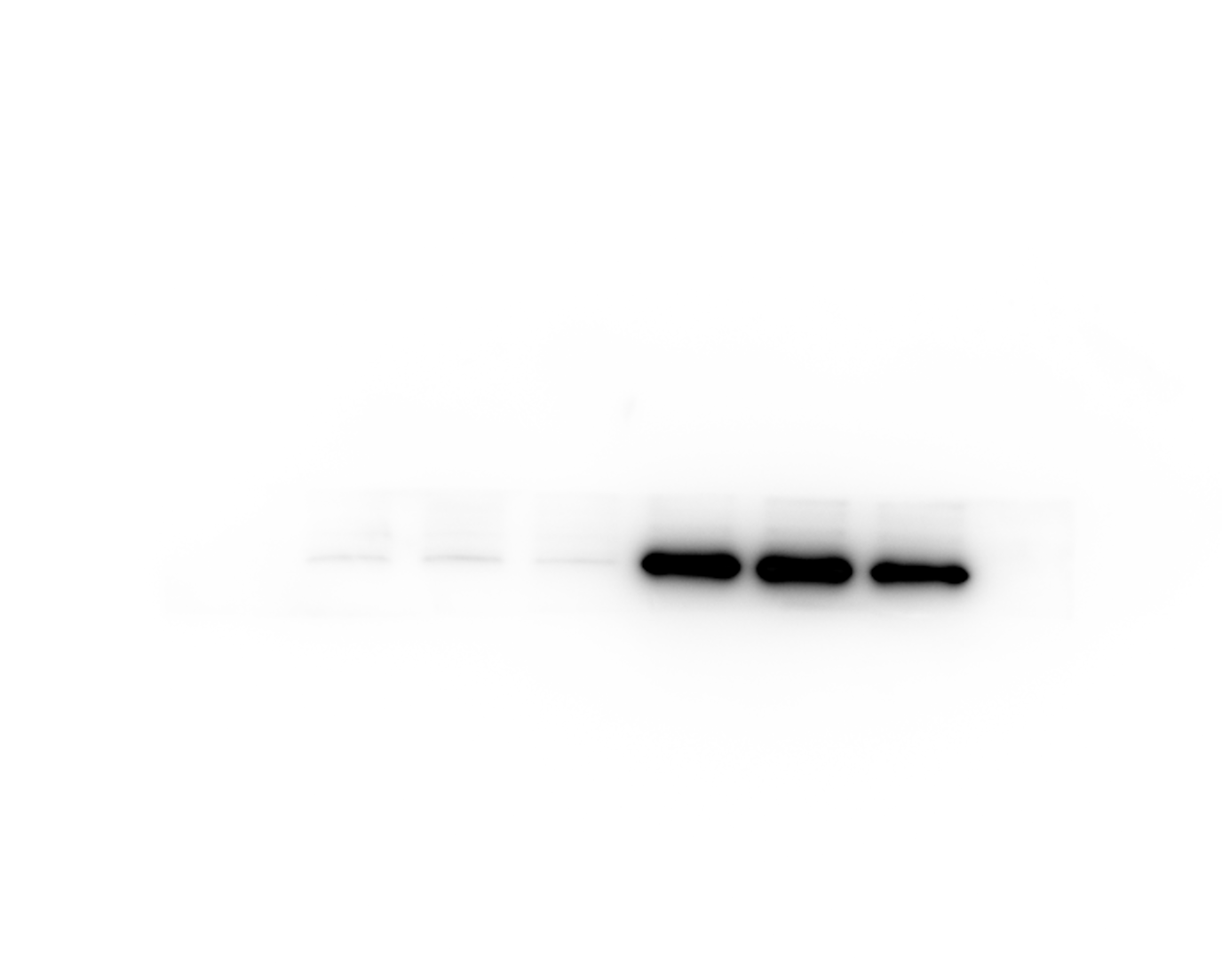


Figure 4H.7721_srebp1


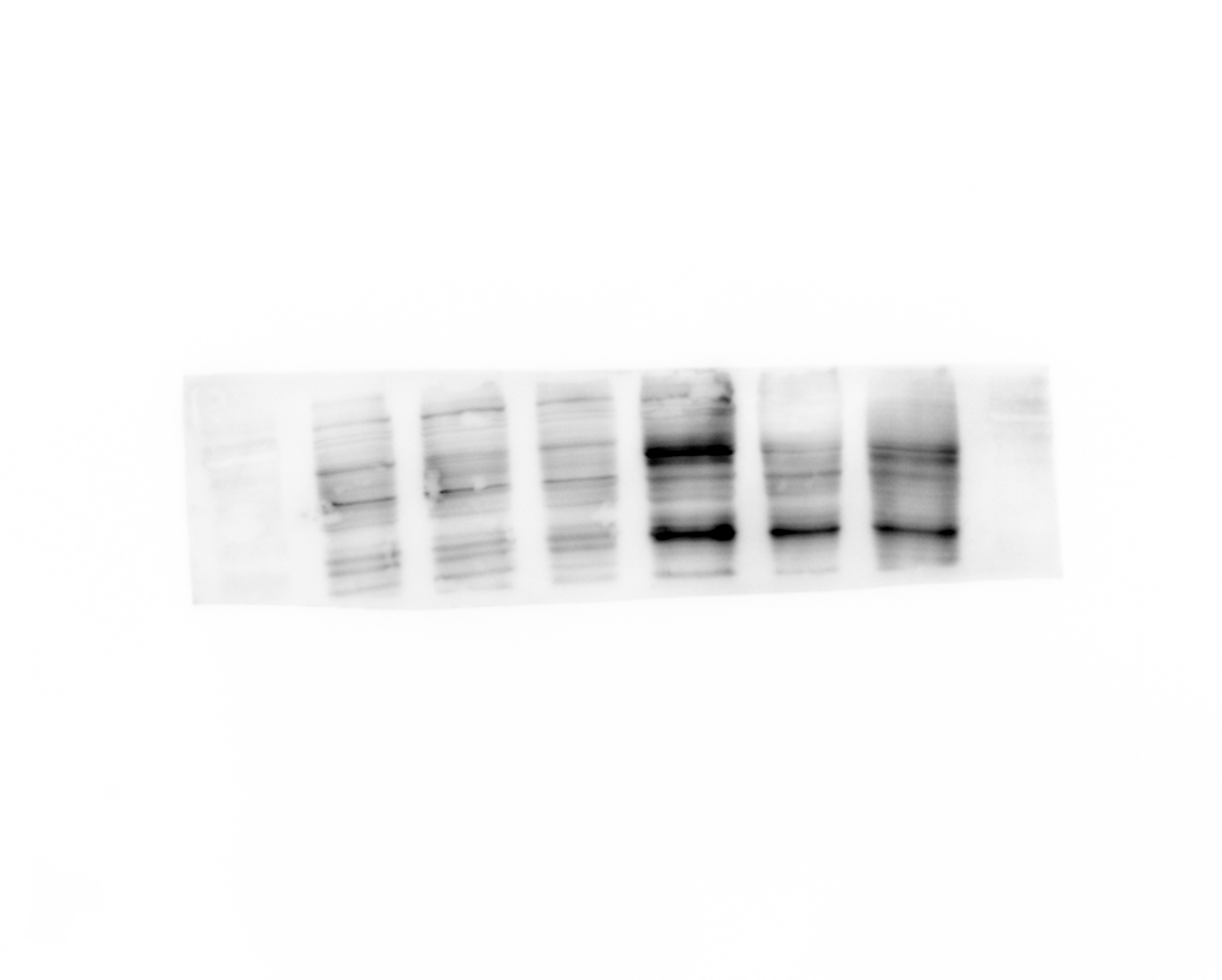


Figure 5D. Huh7_Actin


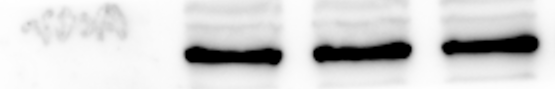


Figure 5D.Huh7_FAds1


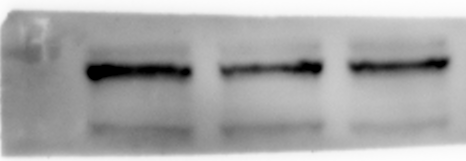


Figure 5D.Huh7_fads2


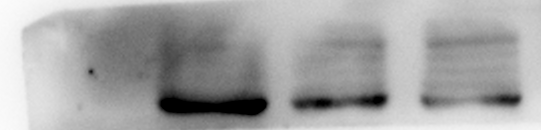


Figure 5D.Huh7_FASN


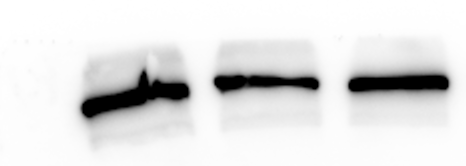


Figure 5D.Huh7_scd1


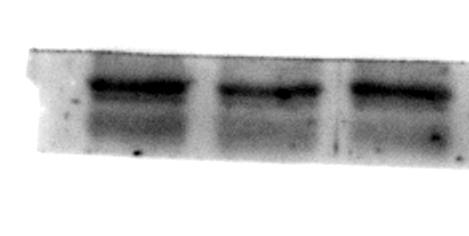


Figure 5B.97H_actin


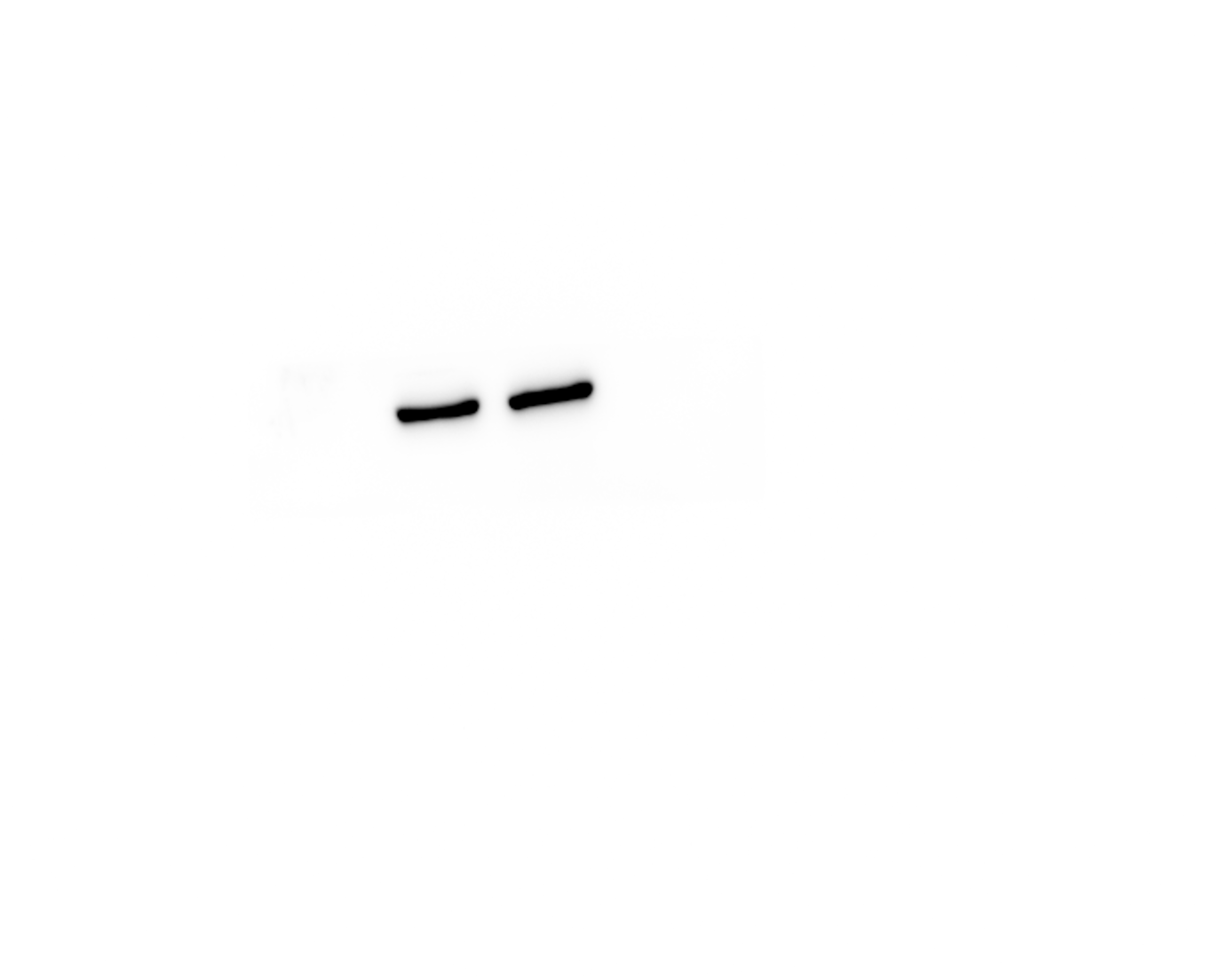


Figure 5B.97H_fads2


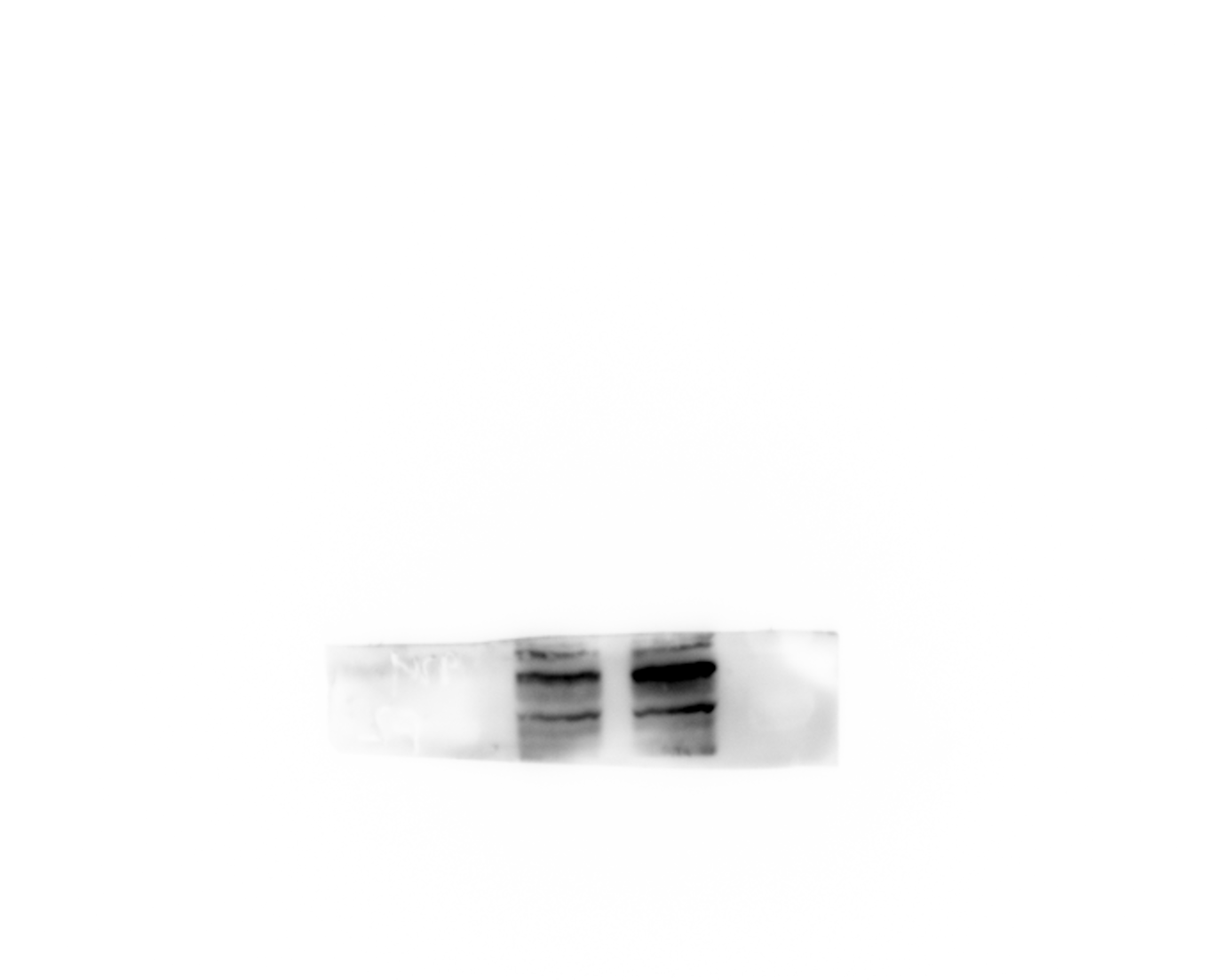


Figure 5B.97H_FASN


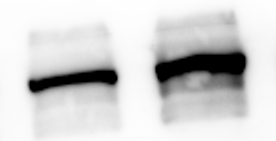


Figure 5B.97H_SCD1


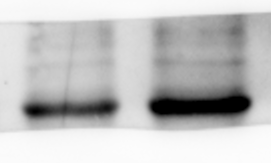


Figure 5B.Hep1_ACTIN


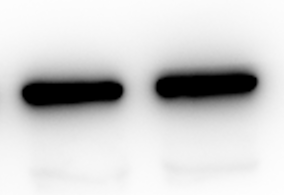


Figure 5B.Hep1_fads1


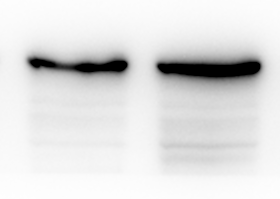


Figure 5B.Hep1_fasn


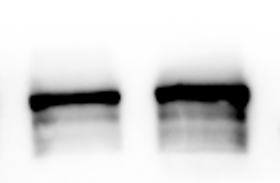


Figure 5B.Hep1_SCD1


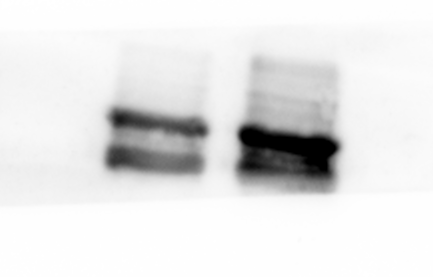


Figure 5D.7721_actin


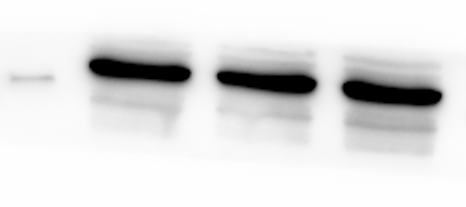


Figure 5D.7721_fads1


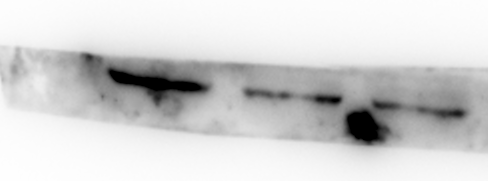


Figure 5D.7721_FADS2


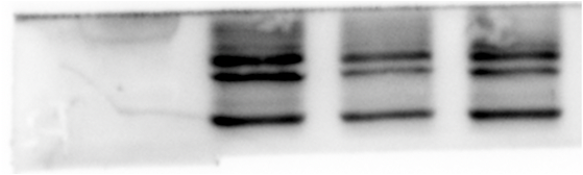


Figure 5D.7721_fasn


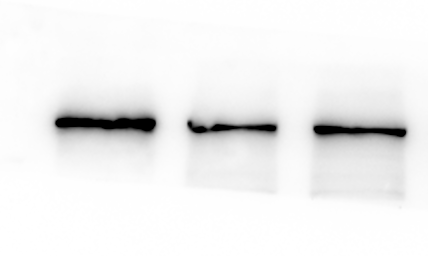


Figure 5D.7721_scd1


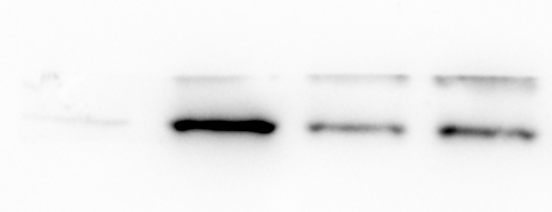


Figure 6A. 97H_fads2


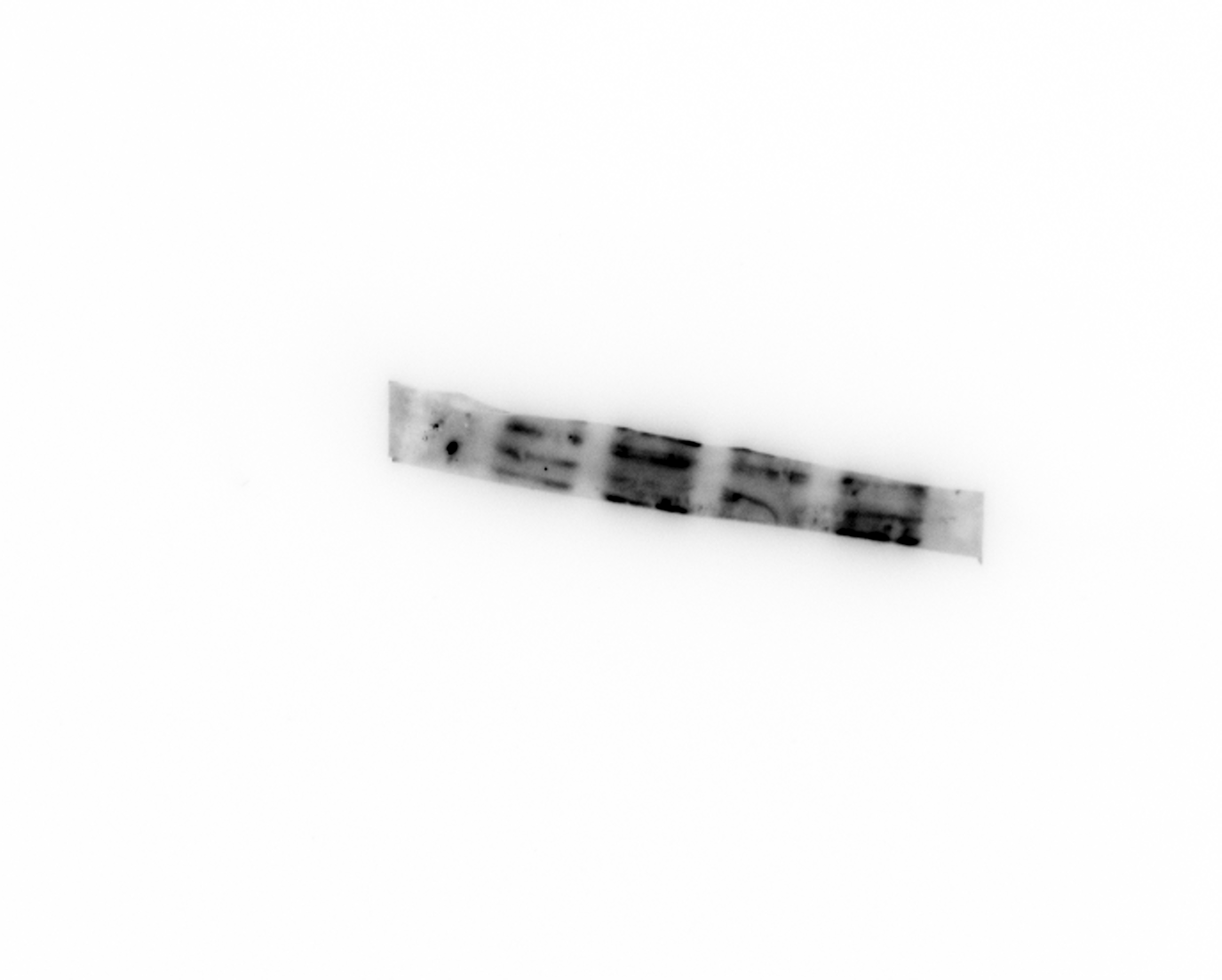


Figure 6A.97H_actin


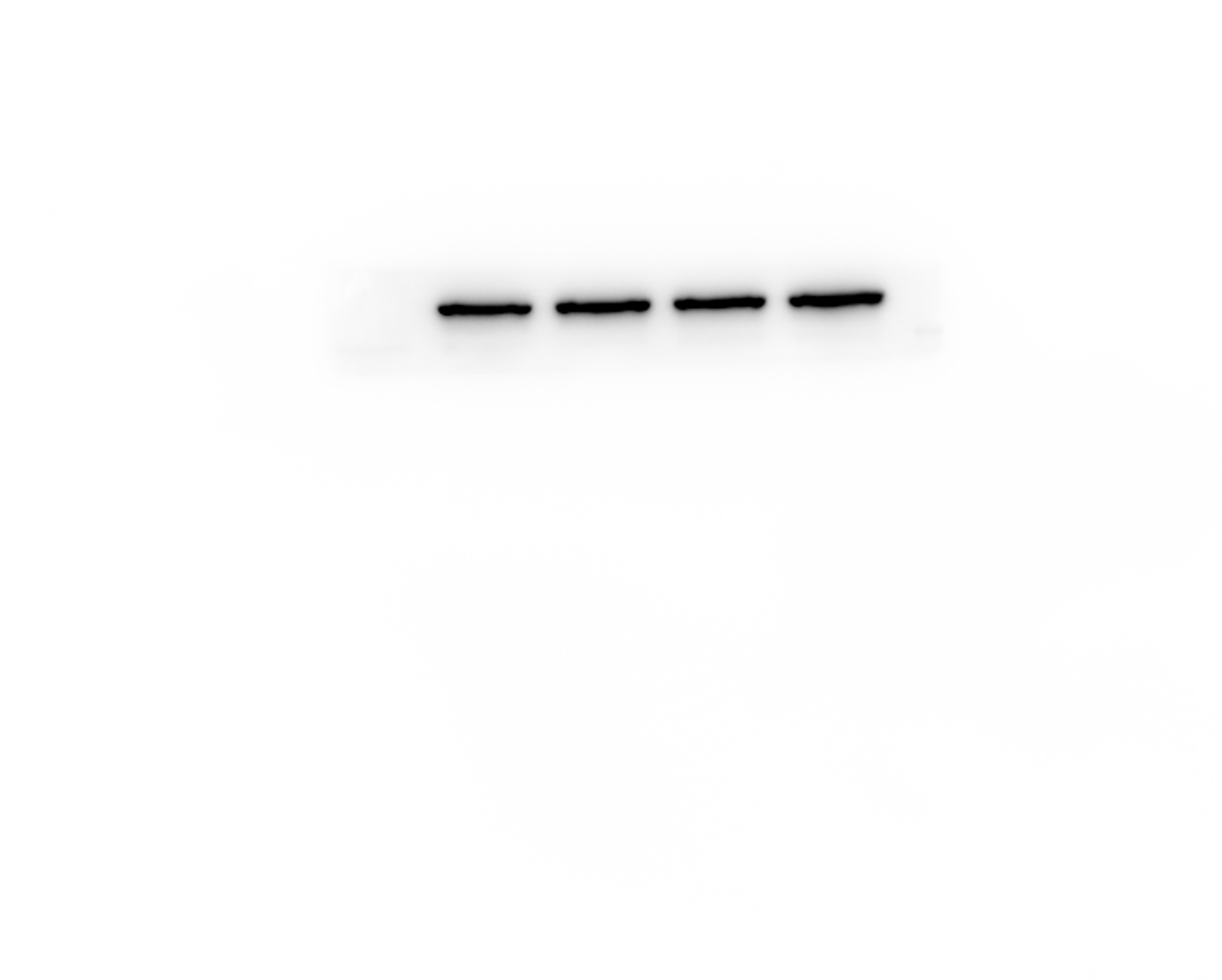


Figure 6A.97H_fasn


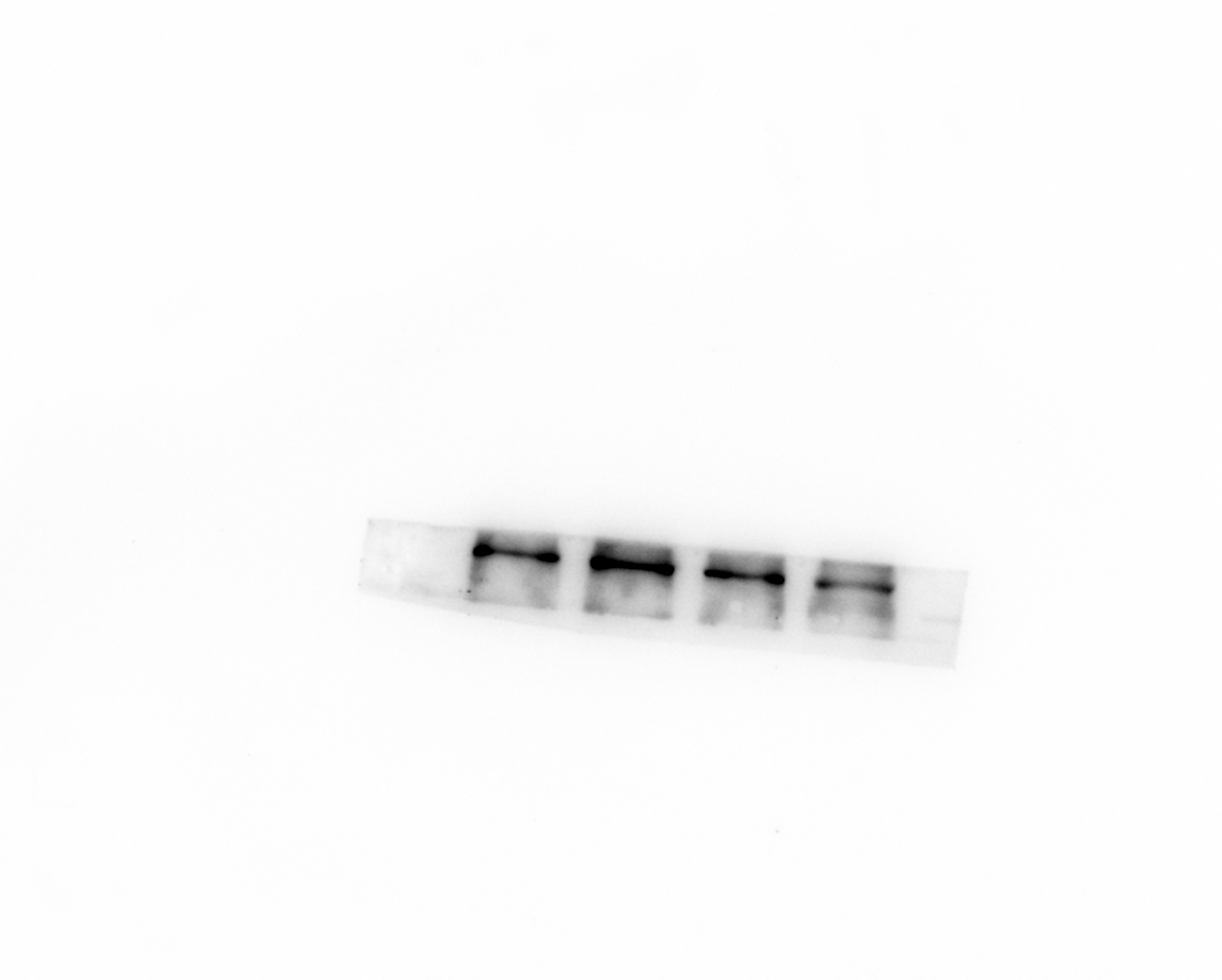


Figure 6A.97H_scd1


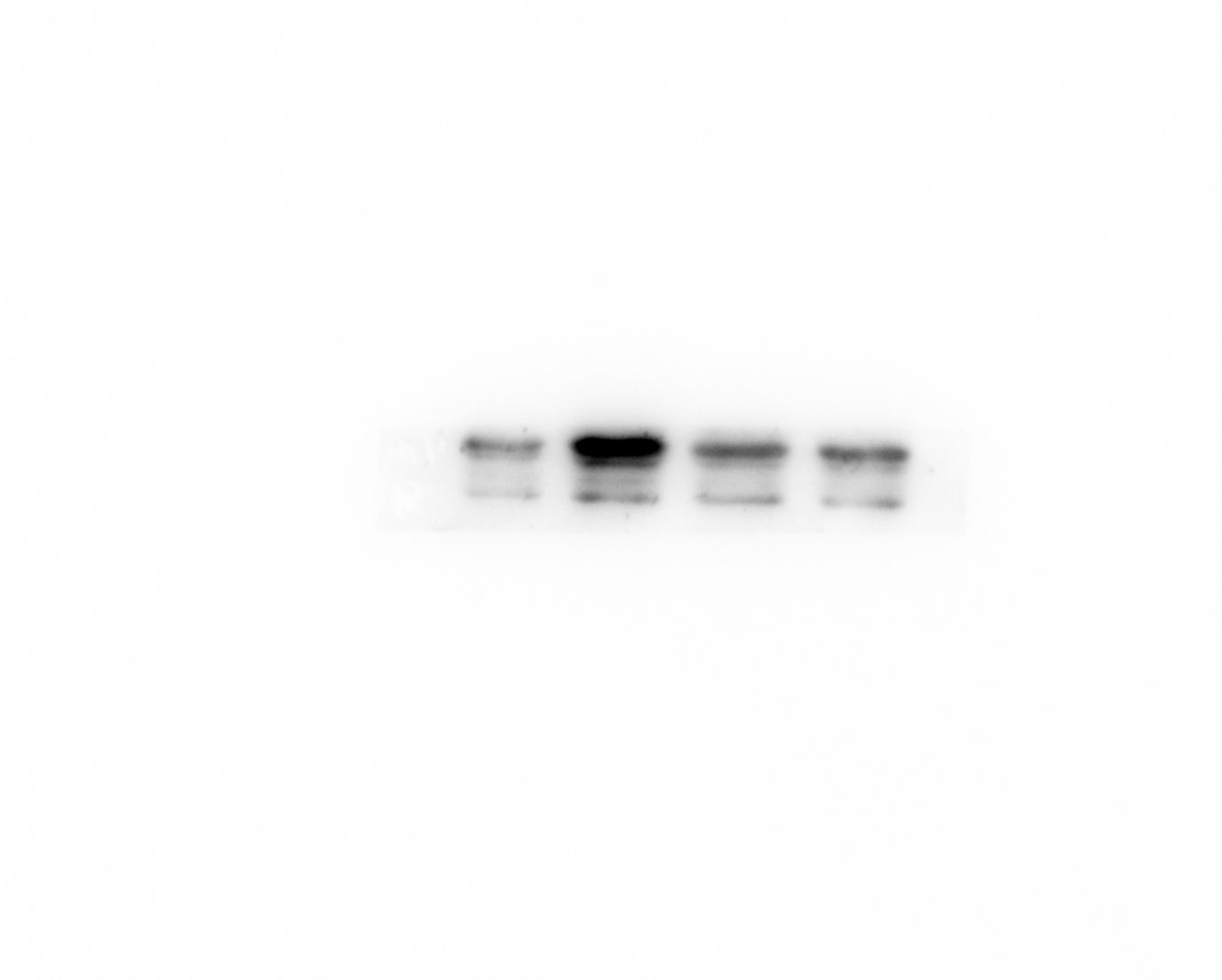


Figure 6A. Hep1_actin


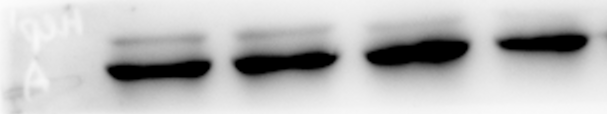


Figure 6A.Hep1_fads1


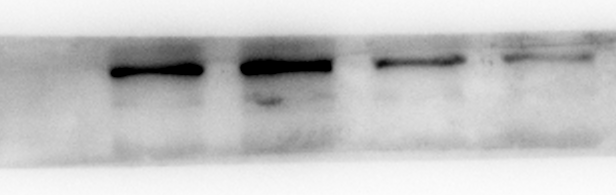


Figure 6A.Hep1_fasn


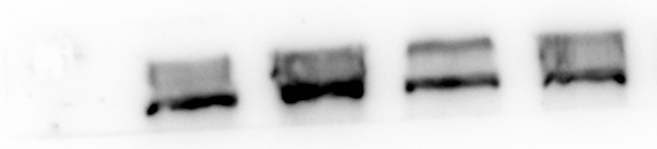


Figure 6A.Hep1_scd1


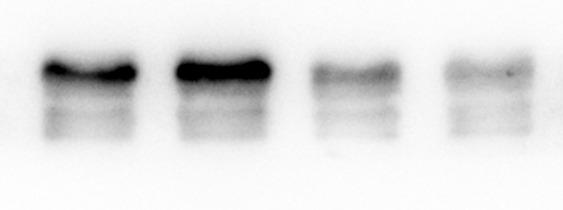

Supplement: Supplementary file 4 — Full and uncropped western blots [file 41420_2022_1213_MOESM4_ESM.docx]
